# Supplementary material for: Bi-allelic variants in three genes encoding distinct subunits of the vesicular AP-5 complex cause hereditary macular dystrophy
Source: Am J Hum Genet. 2025 Mar 12;112(4):808–28. doi: 10.1016/j.ajhg.2025.02.015 (PMC12081239; doi:10.1016/j.ajhg.2025.02.015)
Supplement: Document S1. Figures S1–S5 and financial support [file mmc1.pdf]

The American Journal of Human Genetics, Volume *112*

Supplemental information

Bi-allelic variants in three genes encoding distinct subunits of the vesicular AP-5 complex cause hereditary macular dystrophy

Karolina Kaminska, Francesca Cancellieri, Mathieu Quinodoz, Abigail R. Moye, Miriam Bauwens, Siying Lin, Lucas Janeschitz-Kriegl, Tamar Hayman, Pilar Barberán-Martínez, Regina Schlaeger, Filip Van den Broeck, Almudena Ávila Fernández, Lidia Fernández-Caballero, Irene Perea-Romero, Gema García-García, David Salom, Pascale Mazzola, Theresia Zuleger, Karin Poths, Tobias B. Haack, Julie Jacob, Sascha Vermeer, Frédérique Terbeek, Nicolas Feltgen, Alexandre P. Moulin, Louisa Koutroumanou, George Papadakis, Andrew C. Browning, Savita Madhusudhan, Lotta Gränse, Eyal Banin, Ana Berta Sousa, Luisa Coutinho Santos, Laura Kuehlewein, Pietro De Angeli, Bart P. Leroy, Omar A. Mahroo, Fay Sedgwick, James Eden, Maximilian Pfau, Sten Andréasson, Hendrik P.N. Scholl, Carmen Ayuso, José M. Millán, Dror Sharon, Miltiadis K. Tsilimbaris, Veronika Vaclavik, Hoai V. Tran, Tamar Ben-Yosef, Elfride De Baere, Andrew R. Webster, Gavin Arno, Panagiotis I. Sergouniotis, Susanne Kohl, Cristina Santos, and Carlo Rivolta

Family 13, P16 (52yo), *AP5Z1*

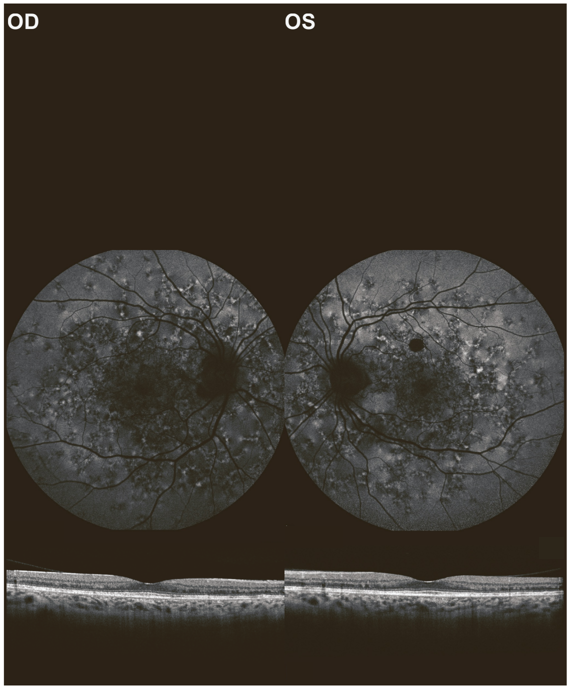

Family 12, P15 (53yo), *AP5Z1*

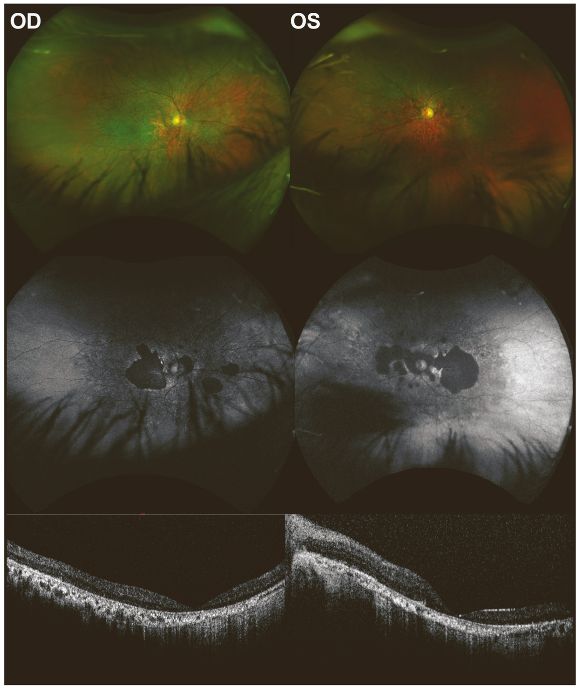

Family 17, P20 (63yo), *AP5M1*

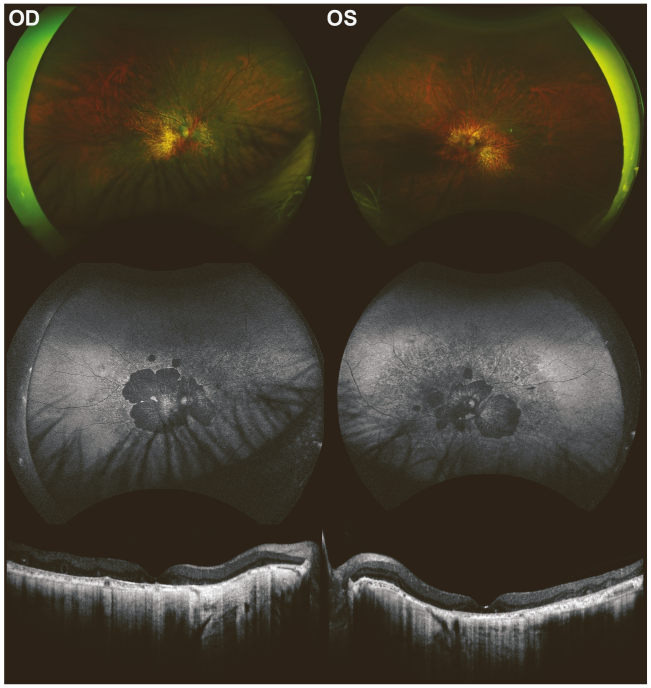

Family 8, P11 (63yo), *AP5Z1*

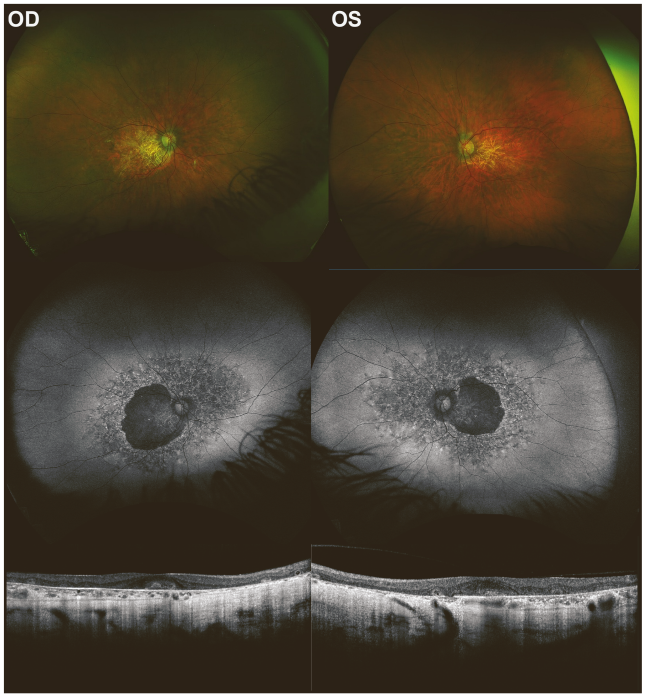

Family 8, P10 (65yo), *AP5Z1*

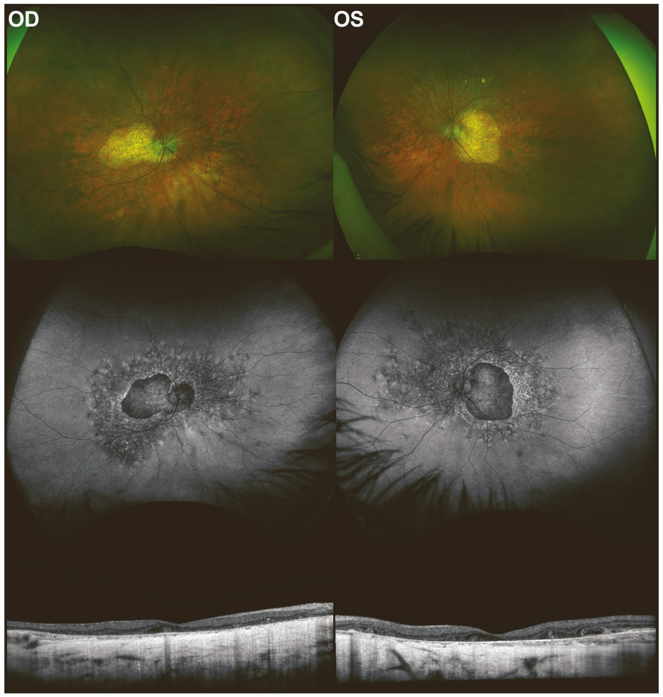

Figure S1: Multimodal retinal imaging of 5 additional individuals from this study. In each panel, the top row shows pseudocolor fundus images, the middle row fundus autofluorescence images (FAF), and the bottom row optical coherence tomography (OCT) scans. For individual P16 from Family 13, color fundus images were not available. This patient presented with a particular phenotype of early maculopathy without atrophy, at the age of 52 years. The other four cases (P15, age 53; P20, age 63; P11, age 63; and P10, age 65) had more advanced central chorioretinal atrophy, regardless of their genotypes. Images are ordered according to the patients' age. OD, right eye; OS, left eye. yo, years old.

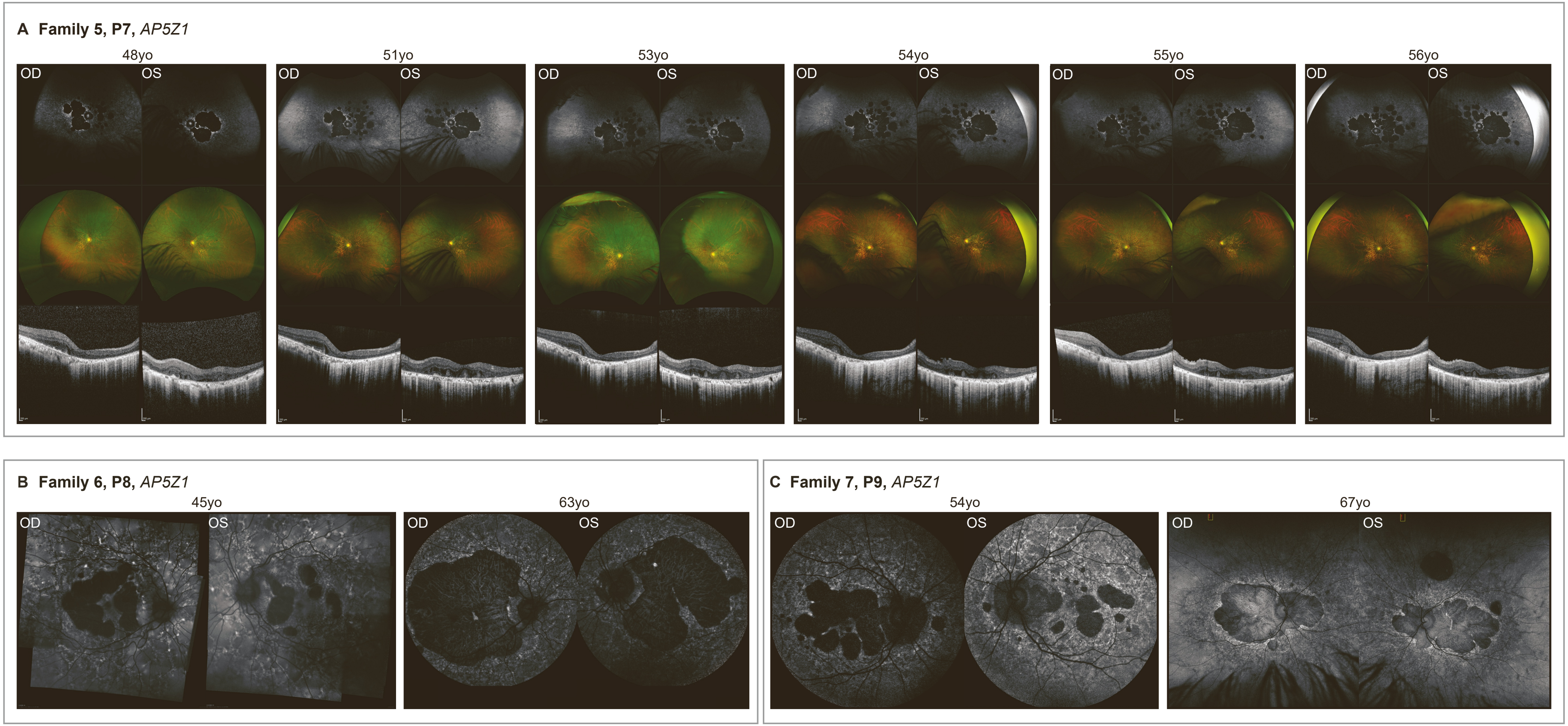

Figure S2: Longitudinal retinal imaging in 3 individuals, showing the progression of their disease. A. In each panel, the top row shows fundus autofluorescence images (FAF), the middle row pseudocolor fundus images, and the bottom row optical coherence tomography (OCT) scans for individual P7 from Family 5, at ages 48, 51, 53, 54, 55, and 56 years. B. FAF images of individual P8 from Family 6, taken at the ages of 45 and 63 years. C. FAF images of individual P9 from Family 7, at the age of 54 years, vs. widefield FAF from the same person at the age of 67 years. OD, right eye; OS, left eye. yo, years old.

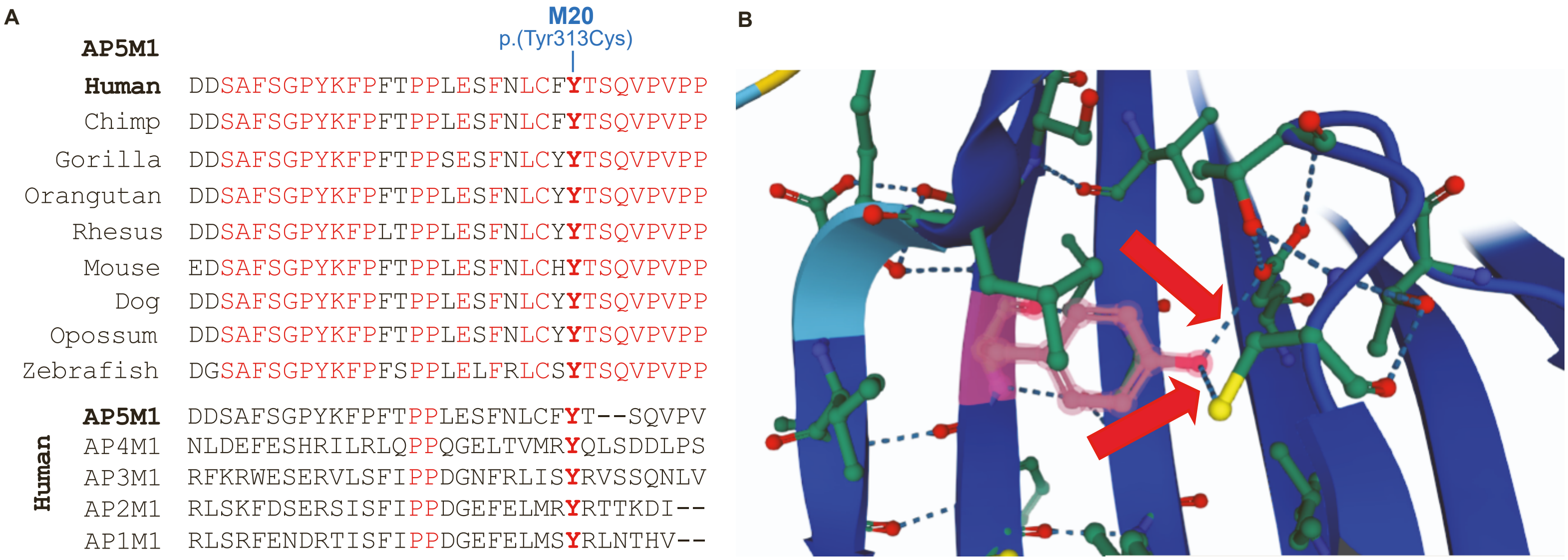

Figure S3: Properties of Tyr313 in AP5M1, affected by the M20 missense change. A. Alignment of part of AP5M1 with its orthologues from various vertebrates, as well as with its human paralogues. The Tyr313 amino acid residue (red, bold) is highly conserved, both across evolution and within the M components of all AP complexes. Other conserved amino acid residues are shown in red. B. Predicted 3D structure of the human AP5M1. The M20 missense [p.(Tyr313Cys)] replaces Tyr313 with a cysteine residue, abolishing the two hydrogen bonds that Tyr313 forms with Glu215 and Cys444 and likely destabilizing the native structure of AP5M1.

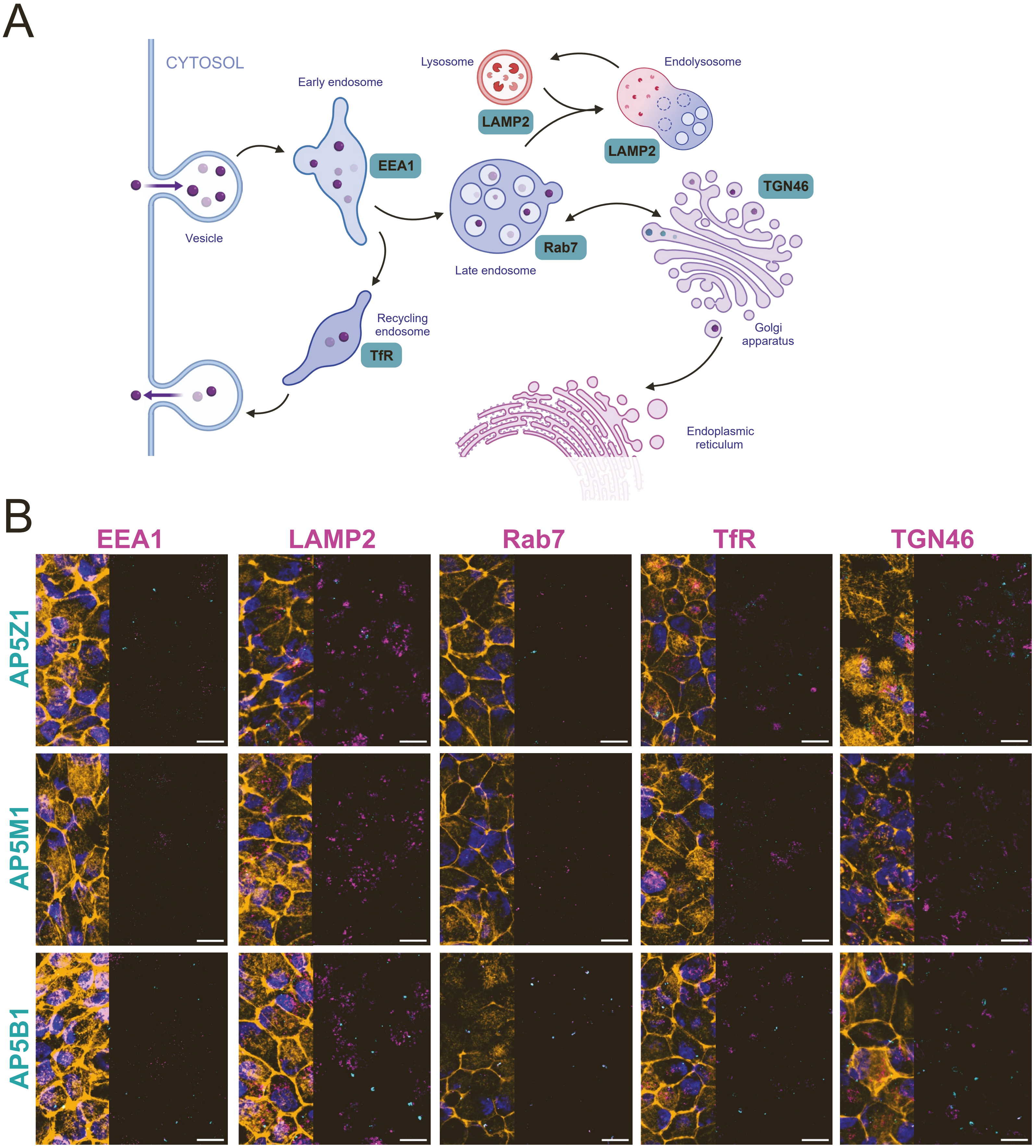

Figure S4: AP5Z1, AP5M1, and AP5B1 staining in human iPSC-RPE cell lines. A. Scheme showing intracellular vesicular transport, including relevant organelles and their associated markers used in this experiment. B. En face imaging of the iPSC-RPE monolayer sections co-stained with respective AP-5 subunits (AP5Z1, AP5M1, AP5B1, cyan) and markers of cellular compartments involved in the endolysosomal processing (magenta). AP-5 components co-localize with Rab7 and partially with TGN46, but not with the other markers. Signals from phalloidin (orange), labeling individual cells' plasma membranes, and DAPI (blue), staining nuclei, are filtered out from the right portion of each panel, to make the other markers more visible. Scale bars: 10 µm.

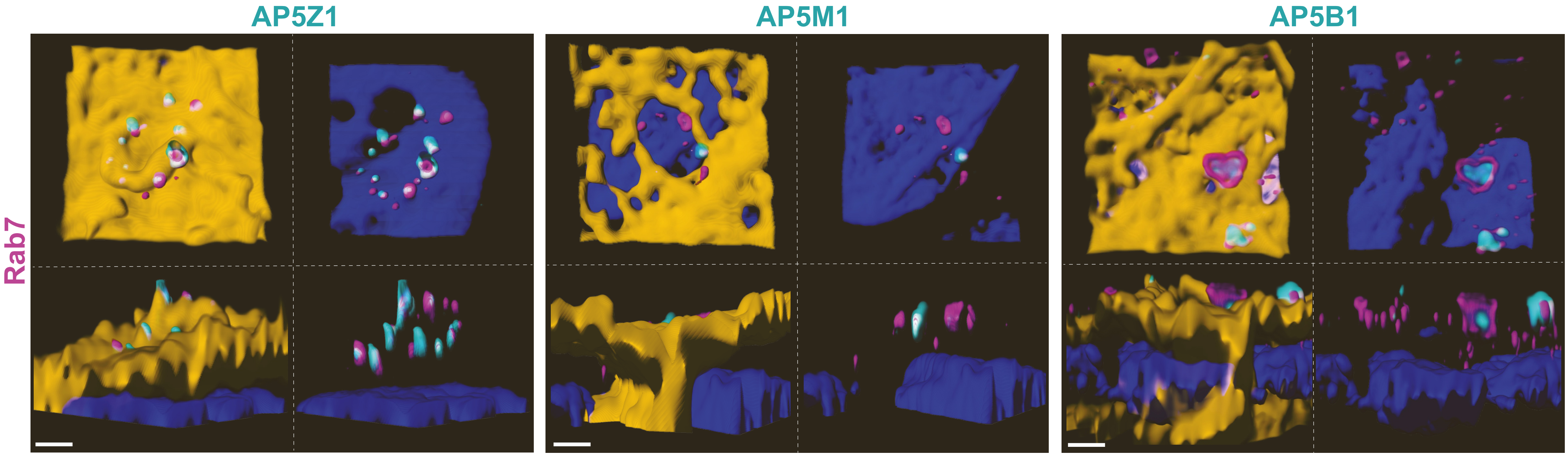

Figure S5: Spatial arrangement of AP5Z1, AP5M1, and AP5B1 in iPSC-RPE. Multi-dimensional visualization of the three studied AP-5 components (cyan) and Rab7-positive vesicles (magenta) in individual iPSC-RPE cells, with regions of their colocalization (white or cyan engulfed by magenta). The top row of each panel shows en-face images of the stained structures, with respect to actin filaments (labeled with **p**halloidin, yellow) and/or nuclei (marked with DAPI, blue). The bottom row of each panel displays cross-sectional views of the same images, illustrating the tubular morphology of these formations and their localization on the apical side of the RPE cells, potentially extending through the microvilli. Scale bars: 1μm.

Financial support

Funding was received from the following agencies: The Swiss National Science Foundation (grant No. 204285, to C.R.); Ministerio de Universidades (Grant No. FPU20/04736, to P.B.M.), Instituto de Salud Carlos III (ISCIII) and co-funded by the European Union (Grant No. CP22/00028 and Grant No. PI22/01371 to GGG, Grant No. PI22/00213, to J.M.M.); Ghent University Special Research Fund (Grant No. BOF20/GOA/023, to E.D.B. and B.P.L.), the European Joint Programme on Rare Diseases (Grant No. EJPRD19-234 Solve-RET, to E.D.B.), Fonds Wetenschappelijk Onderzoek (Grant No. FWO-1802220N, to E.D.B; Grant No. FWO-1803816N, to B.P.L.); the Israel Ministry of Health (Grant No. 3-18611, to T.B.-Y.); the Israel Science Foundation (Grant No. 1778/20- within the Israel Precision Medicine Partnership program, to D.S.); Grant No. PI22/00321, to C.A.; C.A. also receives support from the Centro de Investigación Biomédica en Red Enfermedades Raras (CIBERER, 06/07/0036), and IIS-FJD BioBank (PT13/0010/0012); OAM receives support from the Wellcome Trust and the NIHR Biomedical Research Centre at Moorfields Eye Hospital and the UCL Institute of Ophthalmology; G.A. is supported by a Fight for Sight UK early career investigator award (5045-5046), Moorfields Eye Charity (Stephen and Elizabeth Archer in memory of Marion Woods), NIH-P20GM139769 and the NIHR Biomedical Research Centre at Moorfields Eye Hopsital and the UCL Institute of Ophthalmology; T.B.H. received funding from the German Research Foundation (DFG; Grant No: 418081722 and 433158657) and the European Commission (Recon4IMD - GAP-101080997); P.I.S. receives support from the Wellcome Trust (224643/Z/21/Z) and the NIHR Manchester Biomedical Research Centre (NIHR203308). The views expressed are those of the authors and not necessarily those of the NIHR or the Department of Health and Social Care. E.D.B., B.P.L., S.K. are members of ERN-EYE (Framework Partnership Agreement No 739534-ERN-EYE). C.R., E.D.B., S.K. are members of the ProgRET network (HORIZON-MSCA-2022-DN, No.101120562).
