# Supplementary material for: Bi-allelic variants in three genes encoding distinct subunits of the vesicular AP-5 complex cause hereditary macular dystrophy
Source: Am J Hum Genet. 2025 Mar 12;112(4):808–28. doi: 10.1016/j.ajhg.2025.02.015 (PMC12081239; doi:10.1016/j.ajhg.2025.02.015)
Supplement: Document S2. Article plus supplemental information [file mmc5.pdf]

Bi-allelic variants in three genes encoding distinct subunits of the vesicular AP-5 complex cause hereditary macular dystrophy

Graphical abstract

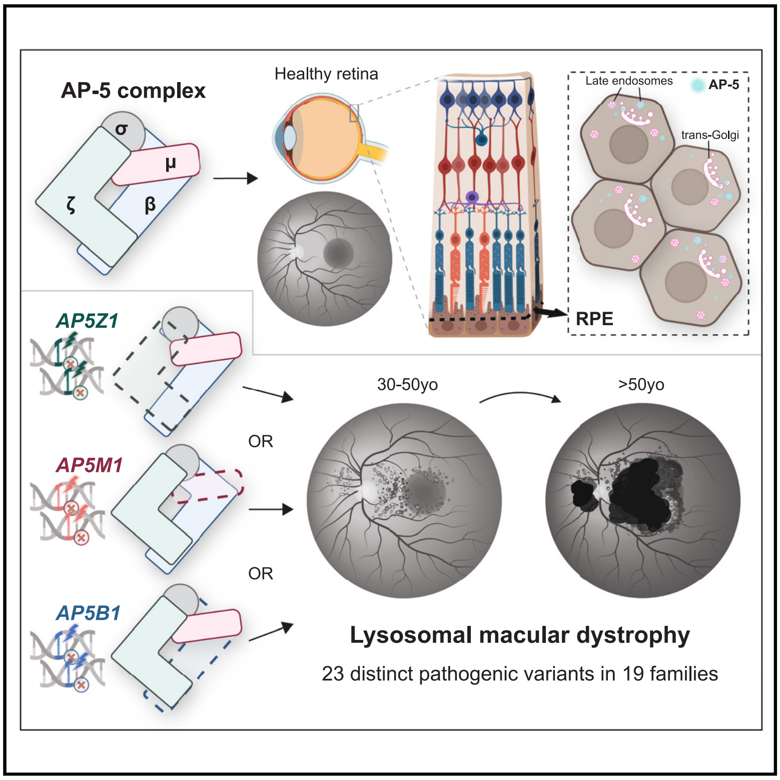

Authors

Karolina Kaminska,
Francesca Cancellieri,
Mathieu Quinodoz, ..., Susanne Kohl,
Cristina Santos, Carlo Rivolta

Correspondence

carlo.rivolta@iob.ch

We discovered genetic changes in three genes (*AP5Z1*, *AP5M1*, and *AP5B1*) that cause macular degeneration, affecting sharp central vision. The proteins encoded by these genes form a complex that helps the cell's recycling system clear waste and keep retinal cells healthy. Some affected individuals also experience additional symptoms beyond vision loss.

Kaminska et al., 2025, The American Journal of Human Genetics *112*, 808–828
April 3, 2025 © 2025 The Author(s). Published by Elsevier Inc. on behalf of American Society of Human Genetics.
https://doi.org/10.1016/j.ajhg.2025.02.015

ARTICLE

Bi-allelic variants in three genes encoding distinct subunits of the vesicular AP-5 complex cause hereditary macular dystrophy

Karolina Kaminska,[1,2] Francesca Cancellieri,[1,2] Mathieu Quinodoz,[1,2,3] Abigail R. Moye,[1,2] Miriam Bauwens,[4,5] Siying Lin,[6,7,8] Lucas Janeschitz-Kriegl,[1,2] Tamar Hayman,[9] Pilar Barberán-Martínez,[10,11] Regina Schlaeger,[12] Filip Van den Broeck,[4,13,14] Almudena Ávila Fernández,[15,16] Lidia Fernández-Caballero,[15,16] Irene Perea-Romero,[15,16] Gema García-García,[10,11,16] David Salom,[16] Pascale Mazzola,[17] Theresia Zuleger,[17] Karin Poths,[17] Tobias B. Haack,[17,18] Julie Jacob,[19] Sascha Vermeer,[20] Frédérique Terbeek,[21] Nicolas Feltgen,[22] Alexandre P. Moulin,[23] Louisa Koutroumanou,[24]

(Author list continued on next page)

Summary

Inherited retinal diseases (IRDs) are a genetically heterogeneous group of Mendelian disorders that often lead to progressive vision loss and involve approximately 300 distinct genes. Although variants in these loci account for the majority of molecular diagnoses, other genes associated with IRD await molecular identification. In this study, we uncover bi-allelic assortments of 23 different (22 loss-of-function) variants in *AP5Z1*, *AP5M1*, and *AP5B1* as independent causes of recessive IRD in members of 19 families from nine countries. Affected individuals, regardless of their genotypes, exhibit a specific form of macular degeneration, sometimes presenting in association with extraocular features. All three genes encode different subunits of the vesicular fifth adaptor protein (AP-5) complex, a component of the intracellular trafficking system involved in maintaining cellular homeostasis and ensuring the proper functioning of lysosomal pathways. The retinal pigment epithelium (RPE), a cellular monolayer located posteriorly to the neural retina, is characterized by intense lysosomal and phagocytic activity. Immunostaining of RPE cells revealed a punctate pattern of AP5Z1, AP5M1, and AP5B1 staining and co-localization with markers of late endosomes and the Golgi, suggesting a role of AP-5 in the normal physiology of this tissue. Overall, the identification of independently acting variants in three distinct proteins within the same macromolecular complex reveals AP-5 as having an important function in the preservation and maintenance of normal macular functions.

Introduction

Inherited retinal diseases (IRDs) are a diverse group of genetic disorders that commonly cause progressive loss of vision due to the degeneration or dysfunction of rod and cone photoreceptors, the light-sensitive neurons of the eye.[1] Although IRDs are monogenic conditions, their genetic architecture is complex and involves alterations in

[1]Institute of Molecular and Clinical Ophthalmology Basel (IOB), 4031 Basel, Switzerland; [2]Department of Ophthalmology, University of Basel, 4031 Basel, Switzerland; [3]Department of Genetics and Genome Biology, University of Leicester, Leicester LE1 7RH, UK; [4]Center for Medical Genetics, Ghent University Hospital, 9000 Ghent, Belgium; [5]Department of Biomolecular Medicine, Ghent University, 9000 Ghent, Belgium; [6]Manchester Centre for Genomic Medicine, Saint Mary's Hospital, Manchester University NHS Foundation Trust, Manchester M13 9WL, UK; [7]Division of Evolution, Infection and Genomics, School of Biological Sciences, Faculty of Biology, Medicine and Health, University of Manchester, Manchester M13 9P, UK; [8]NIHR Biomedical Research Centre, Moorfields Eye Hospital and the UCL Institute of Ophthalmology, London EC1V 9EL, UK; [9]Department of Ophthalmology, Hadassah Medical Center, The Hebrew University of Jerusalem, Jerusalem, Israel; [10]Molecular, Cellular, and Genomic Biomedicine Group, IIS-La Fe, 46012 Valencia, Spain; [11]Joint Unit CIPF-IIS La Fe Molecular, Cellular and Genomic Biomedicine, IIS-La Fe, 46012 Valencia, Spain; [12]Department of Neurology, University Hospital Basel, 4031 Basel, Switzerland; [13]Department of Head & Skin, Ghent University Hospital, 9000 Ghent, Belgium; [14]Department of Ophthalmology, Ghent University Hospital, 9000 Ghent, Belgium; [15]Department of Genetics & Genomics, Instituto de Investigación Sanitaria-Fundación Jiménez Díaz University Hospital, Universidad Autónoma de Madrid (IIS-FJD, UAM), 28040 Madrid, Spain; [16]Center for Biomedical Network Research on Rare Diseases (CIBERER), Instituto de Salud Carlos III, 28029 Madrid, Spain; [17]Institute of Medical Genetics and Applied Genomics, University of Tübingen, 72076 Tübingen, Germany; [18]Centre for Rare Diseases, University of Tübingen, 72072 Tübingen, Germany; [19]Department of Ophthalmology, Universitair Ziekenhuis Leuven (UZ Leuven), 3000 Leuven, Belgium; [20]Center for Human Genetics, University Hospitals Leuven, 3000 Leuven, Belgium; [21]Department of Neurology, Noorderhart Hospital, 3900 Pelt, Belgium; [22]Department of Ophthalmology, University Hospital Basel, 4031 Basel, Switzerland; [23]Jules-Gonin Eye Hospital, Fondation Asile des Aveugles, University of Lausanne, 1004 Lausanne, Switzerland; [24]Medical School, University of Crete, 715 00 Heraklion, Greece; [25]Ophthalmology Department, Royal Victoria Infirmary, Newcastle upon Tyne NE1 4LP, UK; [26]St. Paul's Eye Department, Royal Liverpool University Hospital, Liverpool L7 8XP, UK; [27]Department of Eye and Vision Sciences, Institute of Life Course and Medical Sciences, University of Liverpool, Liverpool L7 8TX, UK; [28]Department of Ophthalmology, Lund University, 223 62 Lund, Sweden; [29]Department of Medical Genetics, Hospital Santa Maria, Unidade Local de Saúde de Santa Maria, 1649-035 Lisbon, Portugal; [30]Medical Genetics University Clinic, Faculty of Medicine, University of Lisbon, 1649-028 Lisbon, Portugal; [31]Department of Ophthalmology, Instituto de Oftalmologia Dr Gama Pinto (IOGP), 1169-019 Lisbon, Portugal; [32]University Eye Hospital, Center for Ophthalmology, University of Tübingen, 72076 Tübingen, Germany; [33]Institute for Ophthalmic Research, Center for Ophthalmology, University of Tübingen, 72076 Tübingen, Germany; [34]Division of Ophthalmology, Children's Hospital of Philadelphia, Philadelphia, PA 19104, USA; [35]UCL

(Affiliations continued on next page)

© 2025 The Author(s). Published by Elsevier Inc. on behalf of American Society of Human Genetics.
This is an open access article under the CC BY license (http://creativecommons.org/licenses/by/4.0/).

George Papadakis,[24] Andrew C. Browning,[25] Savita Madhusudhan,[26,27] Lotta Gränse,[28] Eyal Banin,[9] Ana Berta Sousa,[29,30] Luisa Coutinho Santos,[31] Laura Kuehlewein,[32] Pietro De Angeli,[33] Bart P. Leroy,[4,13,14,34] Omar A. Mahroo,[8,35,36] Fay Sedgwick,[37] James Eden,[37] Maximilian Pfau,[1,22] Sten Andréasson,[28] Hendrik P.N. Scholl,[38,39,40] Carmen Ayuso,[15,16] José M. Millán,[10,11,16,41] Dror Sharon,[9] Miltiadis K. Tsilimbaris,[24] Veronika Vaclavik,[23] Hoai V. Tran,[23,42] Tamar Ben-Yosef,[43] Elfride De Baere,[4,5] Andrew R. Webster,[8,35] Gavin Arno,[8,35,44] Panagiotis I. Sergouniotis,[6,7,45] Susanne Kohl,[33] Cristina Santos,[31,46] and Carlo Rivolta[1,2,3,*]

genes that are usually linked to essential processes of various retinal cells, especially the photoreceptors and cells of the retinal pigment epithelium (RPE).

Based on the cell types that are primarily compromised and the pattern of retinal degeneration, IRDs can be classified into several clinical categories, including retinitis pigmentosa (RP), cone-rod dystrophy (CRD), or macular dystrophy (MD). The latter primarily affects the macula, the central region of the retina that is rich in cone photoreceptors and is responsible for high-acuity vision, required for visual tasks such as reading, watching movies, or recognizing faces. Individuals with MD typically experience initial symptoms such as central vision loss, photophobia, metamorphopsia (visual distortion), or scotomas (blind spots).[2] Out of the ~300 genes associated with IRDs and reported in the RetNet database (http://retnet.org, as of August 2024), fewer than 20 have been linked to MD and only four to recessive MD.

The inheritance patterns of IRDs reflect their genetic complexity and include autosomal recessive (the most prevalent form), autosomal dominant, as well as X-linked and mitochondrial inheritance.[3] Understanding the genetic etiology of IRD is crucial for the accurate diagnosis of affected individuals, for the genetic counseling of families, and for the development of future targeted therapies. The advent of next-generation sequencing technologies and the development of novel *in silico* bioinformatics tools have recently provided significant advancements in the field of IRD molecular genetics.[4] Despite this substantial progress, a considerable proportion of individuals with IRDs remain without a definitive genetic diagnosis, the current molecular diagnostic rate varying between 50% and 80%, mostly as a function of the population studied.[3–9] This missing heritability can be attributed to several factors, including technical limitations of current methods (such as the detection of deep intronic or structural variants),[10] challenges in prioritizing certain DNA changes (such as synonymous and non-coding variants), and the existence of still undiscovered disease-associated genes and pathways.[11–14]

Lysosomal storage diseases (LSDs) result from Mendelian variants in more than 50 different genes and are ultimately linked to the impairment of lysosomal function and the abnormal degradation of cellular waste products, which, in turn, lead to the progressive accumulation of metabolic by-products and subsequent cellular damage.[15] Individuals with LSDs typically manifest multi-organ signs and symptoms such as developmental delay, organomegaly, central nervous system deficits, and, in some instances, retinopathy.[15] Some genes implicated in LSDs, such as *CLN3* (MIM: 607042),[16] *HGSNAT* (MIM: 610453),[17] and *MFSD8* (MIM: 611124)[18,19] have also been associated with non-syndromic IRDs, where the buildup of autofluorescent waste material impairs normal cellular function and contributes to retinal degeneration.[20]

The fifth adaptor protein (AP-5) complex plays a crucial role in intracellular trafficking, and, in particular, in the sorting and transport of proteins within the late endosome-to-Golgi retrieval network.[21] This process is essential for maintaining cellular homeostasis and ensuring the proper functioning of lysosomal pathways.[21] Moreover, the AP-5 complex is thought to be involved in the recovery of lysosomes from endolysosomes, a fundamental process for the maintenance of the balance between different stages of endosomal and lysosomal maturation.[22] AP-5 is composed of four subunits, zeta, mu, beta, and sigma, encoded by the genes *AP5Z1* (MIM: 613653), *AP5M1* (MIM: 614368), *AP5B1* (MIM: 614367), and *AP5S1* (MIM: 614824), respectively.[23] Notably, pathogenic variants in only one of them, *AP5Z1*, have been associated with recessive hereditary spastic paraplegia, type 48 (SPG48, MIM: 613647),[24] a neurological disorder characterized by progressive weakness and spasticity of the lower limbs. This condition can be classified as a form of LSD due to the accumulation of aberrant endolysosomes in cells lacking functional AP-5.[25] However, the specific function and pathophysiology of the other components of the AP-5 complex remain elusive.

In this study, we identify bi-allelic variants in genes encoding three of the four subunits of the AP-5 complex (*AP5Z1*, *AP5M1*, and *AP5B1*) as a previously unreported

Institute of Ophthalmology, University College London, London EC1V 9EL, UK; [36]Department of Ophthalmology, St Thomas' Hospital, London SE1 7EH, UK; [37]Eye Team, North West Genomic Laboratory Hub, St Mary's Hospital, Manchester M13 9WL, UK; [38]Department of Clinical Pharmacology, Medical University of Vienna, 1090 Vienna, Austria; [39]Pallas Kliniken AG, Pallas Klinik Zürich, 8005 Zürich, Switzerland; [40]European Vision Institute, 4056 Basel, Switzerland; [41]University and Polytechnic La Fe Hospital of Valencia, 46026 Valencia, Spain; [42]Centre for Gene Therapy and Regenerative Medicine, King's College London, London WC2R 2LS, UK; [43]The Ruth & Bruce Rappaport Faculty of Medicine, Technion-Israel Institute of Technology, Haifa 31096, Israel; [44]Division of Research, Greenwood Genetic Center, Greenwood, SC 29646, USA; [45]Manchester Royal Eye Hospital, Manchester University NHS Foundation Trust, Manchester M13 9WL, UK; [46]iNOVA4Health, NOVA Medical School, Faculdade de Ciências Médicas, NMS, FCM, Universidade NOVA de Lisboa, 1099-085 Lisbon, Portugal
*Correspondence: carlo.rivolta@iob.ch
https://doi.org/10.1016/j.ajhg.2025.02.015.

cause of IRD, with or without extraocular features, in 22 subjects from 19 unrelated families from nine different countries. All affected individuals exhibited a specific form of MD, which we propose to classify as a distinct subtype of IRD: lysosomal macular dystrophy.

Material and methods

Families and DNA samples

This study adhered to the tenets of the Declaration of Helsinki and was approved by the Ethics Committees of the respective Institutions (Comissão de Ética para a Saúde do Instituto de Oftalmologia Dr. Gama Pinto, Cantonal Committee of Canton Vaud for Research Activities on Human Subjects, Ethikkommission Nordwest- und Zentralschweiz, Ethics Board of the Medical Faculty of the University Tübingen, Ethical Committee for Medical Research at Lund University, Ethic Committee of the Hadassah Medical Center, Research Ethics Committee of the Fundación Jiménez Díaz Hospital, Comité de Ética del Instituto de Investigación Sanitaria La Fe, Commissie voor Medische Ethiek UZ Gent, London – Camden & Kings Cross Research Ethics Committee, Wales REC5, the North West of England Research Ethics Committee, and Ethics Committee of Technion – Israel Institute of Technology). Written informed consent was obtained from all individuals or their legal guardians prior to their inclusion in this study. All subjects underwent ophthalmological evaluation. Available records including clinical description of slit-lamp examination, best-corrected visual acuity, perimetry, full-field electroretinography (ERG) and multimodal fundus imaging with retinography, autofluorescence (FAF), optical coherence tomography (OCT), and fluorescein angiography were analyzed.

DNA was obtained from whole-blood or saliva samples.

Whole-exome sequencing, whole-genome sequencing, and data analysis

Whole-exome sequencing (WES) was performed according to protocols that were specific to each of the participating Institutions but had nonetheless a common structure, described in detail previously.[8,26–28]

Whole-genome sequencing (WGS) was performed either as part of the Genomics England 100,000 Genomes project or the UK NHS Genomic Medicine Service.[29,30] Bioinformatics analysis and interpretation of results were initially performed using a clinical pipeline focusing on protein-altering variants within the R32 PanelApp Retinal Disorders gene panel.[31] Additional WGS was done at the Institute for Medical Genetics and Applied Genomics at the University Hospital Tübingen, Germany, as described previously.[9]

All variants were validated using VariantValidator,[32] described in accordance with the Human Genome Variation Society (HGVS) nomenclature,[33] and classified using American College of Medical Genetics and Genomics (ACMG) criteria.[34,35] Frequency of DNA variants in the general population was downloaded from the gnomAD database (v.2.1.1).[36] We refrained from using later versions of this database for variant filtering, since they may contain genotypes from individuals with Mendelian conditions (https://clinicalgenome.org/docs/clingen-guidance-to-vceps-regarding-the-use-of-gnomad-v4, v.2.0). Homozygous calls were confirmed to be bi-allelic by the direct analysis of BAM files, IGV, and/or OFF-PEAK.[37,38]

Structural large alterations were detected in short-read WGS data using the Canvas[39] and/or Manta[40] algorithms. A targeted single-nucleotide polymorphism (SNP) array was used to validate the M10 variant (see below). Infinium GSA-24+ v.3.0 arrays (Illumina) were used to test the template DNA, prepared according to the manufacturer's protocol. Alterations were visualized and analyzed using the NxClinical 6.2 and FASST2 CNV calling algorithm software (BioDiscovery).

Protein alignment and structure predictions were performed by using T-Coffee,[41] Expresso,[42] and AlphaFold.[43]

Disease prevalence estimation

To estimate the prevalence of AP5-related diseases, we utilized the GeniE tool (https://genie.broadinstitute.org/) by using manually curated lists of variants extracted from gnomAD v.2.1.1.[36] As per the PVS1 (pathogenic very strong 1) criterion of the ACMG guidelines,[34,35] we considered true loss-of-function alleles all variants labeled as "LoF" by gnomAD, with the exception of those located in the last exon of the genes (for *AP5Z1* and *AP5M1*) or removing more than 10% of the protein (for *AP5B1* and *AP5S1*, which have very large terminal exons). Additionally, the c.938A>G (p.Tyr313Cys) variant for *AP5M1* was curated manually. All selected variants were verified to affect their relevant transcripts.

Targeted Sanger sequencing

DNA sequencing using the Sanger method was performed to validate short-read sequencing findings and to perform intrafamilial segregation analyses, where appropriate. Primer3Plus[44] was used to design primers for PCR, performed using the GoTaq polymerase (Promega) or FIREPol DNA polymerase (Solis BioDyne) and ~2 ng of template DNA, according to the manufacturers' protocols. All PCR products were treated with ExoSAP-IT (Thermo Fisher) or Illustra ExoProStar (Cytiva), and Sanger sequencing was performed by Microsynth (Balgach, Switzerland) or by StabVida (Madrid, Spain). Sequences were visualized and compared to the human reference sequence (Ensembl,[45] GRCh37) with the CLC Genomics Workbench 12 software (QIAGEN). Sequences of primers and PCR conditions are available upon request.

RNA analysis

RNA analysis was performed to test the effects on splicing of the variant c.1595G>T (GenBank: NM_014855.3) (M2) in *AP5Z1*, detected in multiple individuals. Three milliliters of peripheral blood were collected in Tempus Blood RNA tubes (Applied Biosystems) from P1, an affected person who was heterozygous for the studied variant, as well as from a control individual. Total leukocyte RNA was extracted with the Tempus Spin RNA Isolation Kit (Applied Biosystems) according to the manufacturer's instructions. RNA from healthy retina was obtained commercially (Clontech, Takara). cDNA was synthesized from 2 μg of RNA with the MultiScribe reverse transcriptase from the High-Capacity cDNA Reverse Transcription Kit (Applied Biosystems) according to the manufacturer's instructions. A negative control for the RT-PCR experiment (RT(−)) was prepared by removing reverse transcriptase from one of the RNA samples from the control donor.

RT-PCR amplification was performed using GoTaq G2 DNA polymerase and the provided reaction buffer (Promega) by using primers 5′-CACCTCAGCACCCTCAGATT-3′ (CR-8319) and 5′-CCTGGGGATCAGATCTTGG-3′ (CR-8320) lying in the *AP5Z1* exon 10 and the junction between exons 16 and 17, respectively, according to standard cycling conditions. The resulting PCR

products were resolved on a 1.5% agarose gel. Amplicons were either directly sequenced (as described above) or purified using NucleoSpin Gel and PCR Clean-up Kit (Macherey-Nagel), prior to being subcloned into pGEM-T Easy vectors (Promega). Individual clones were then sequenced using the primers mentioned above.

Immunostaining of human RPE explants

Human RPE flat mounts were collected and stored in Ames' medium (Sigma-Aldrich, cat. #A1420) for 4 h following enucleation from a patient (a 67-year-old woman with uveal melanoma) and subsequently fixed using 4% paraformaldehyde (PFA) in 1× phosphate-buffered saline (PBS, Merck) for 2 h. The tissue was then incubated in 30% sucrose in PBS overnight at 4°C. For immunofluorescence staining, tissues were blocked for 1 h in block buffer (5% bovine serum albumin, 10% normal goat serum, 0.4% fish skin gelatin, and 0.1% Triton X-100 in PBS). Tissues were incubated with primary antibodies overnight at 4°C, followed by three 5-min washes in PBS, prior to a 1-h incubation with the secondary antibodies. After three more washes in PBS, tissues were mounted on slides with ProLong Gold Antifade Mountant (Thermo Fisher) and #1.5 glass coverslips. Immunofluorescence staining was performed with the following primary antibodies, diluted in the block buffer: anti-AP5Z1 (Invitrogen, cat. #PA5-57368, 1:50 dilution), anti-AP5M1 (Invitrogen, cat. #PA5-63174, 1:30 dilution), and anti-AP5B1 (Invitrogen, cat. #PA5-64344, 1:100 dilution). The secondary antibody used was Alexa Fluor 555 F(ab′)2 goat anti-rabbit immunoglobulin G (IgG) (Invitrogen, cat. #A-21430, 1:500 dilution). DAPI (Thermo Fisher, cat. #D1306, 1:500 dilution) was used for nuclear staining. Phalloidin-iFluor 647 conjugate (Abcam, cat. #ab176759, 1:1,000 dilution) was used to stain actin filaments. The specificity of the staining was confirmed by incubation with the secondary antibody only. Imaging was performed with an Olympus scanning confocal microscope, using a 63× oil objective.

Induced pluripotent stem cell-derived RPE cell culture and immunostaining

Human induced pluripotent stem cell-derived RPE (iPSC-RPE) cells were differentiated from the iPS(IMR90)-4-DL-01 line following previously established protocols.[46,47] After culturing as a monolayer in transwells for 1 week, cells were fixed with 4% PFA in 1× PBS for 20 min and then stored in PBS at 4°C. For immunofluorescence staining, membrane inserts were removed from transwells, sectioned, and stained as described above for human RPE explants. The iPSC-RPE sections were then mounted on slides with the ProLong Glass Antifade Mountant (Thermo Fisher) and covered with #1.5 glass coverslips. Immunofluorescence staining was performed with the following primary antibodies, diluted in the block buffer: anti-AP5Z1 (as above), anti-AP5M1 (as above, 1:50 dilution), anti-AP5B1 (as above), anti-EEA1 (BD Biosciences, cat. #610456, 1:100 dilution), anti-LAMP2 (Abcam, cat. #ab25631, 1:100 dilution), anti-TfR (Invitrogen, cat. #13–6800, 1:100 dilution), anti-Rab7 (Abcam, cat. #ab50533, 1:100 dilution), and anti-TGN46 (Sigma-Aldrich, cat. #sab4200355, 1:100 dilution). Secondary antibodies used were Alexa Fluor 488 F(ab′)2 goat anti-mouse IgG (Invitrogen, cat. #A-11017) and Alexa Fluor 594 F(ab′)2 goat anti-rabbit IgG (Invitrogen, cat. #A-21428), both at a 1:500 dilution. DAPI (as above) was used for nuclear staining. Phalloidin-iFluor 647 conjugate (as above) was used to stain actin filaments. The specificity of the staining was confirmed by incubation with the secondary antibody only. Imaging was performed with an Olympus scanning confocal microscope, using a 63× oil objective. For higher resolution, imaging was performed on a Leica STELLARIS 8 Falcon point scanning confocal microscope, using an HC PL APO 63×/1.40 objective. Lightning processing was performed immediately after imaging on LASX software.

Results

Clinical characteristics of the affected study subjects

A total of 22 affected individuals from 19 unrelated families (Figure 1) were ascertained by specialized ophthalmological examinations. In all instances, the disease appeared to be transmitted as an autosomal recessive trait, since the disorder manifested in individuals of both sexes, all parents of affected individuals were healthy by history, and, in some cases, probands had siblings presenting with the same condition. All subjects exhibited ophthalmic features characteristic of progressive MD (Tables 1 and S1), with central vision symptoms having their onset on average during the 5th decade of life (45 years, range 30–57 years, Table S1). In three subjects (P14, P16, and P22), the disease presented as flecked maculopathy without extensive atrophy (Figures 2A and S1). Two probands of an intermediate stage (P5 and P18) exhibited outer retinal and RPE atrophy alongside some remaining flecks (Figure 2B). All affected individuals who were in the 6th decade of life and beyond demonstrated a variable area of central chorioretinal atrophy extending centrifugally (Figures 2C and S1), characterized by complete RPE and outer retinal loss on OCT, as well as complete loss of short-wavelength autofluorescence signal. This atrophy was primarily macular and nasal. Nine individuals presented a characteristic reticular pigment clumping pattern in the peripheral retina (Table S1), one eye showed an inactive choroidal neovascular membrane (P18, Figure 2B), and 12 individuals with advanced disease presented outer retinal tubulations (Table S1). Longitudinal data available for at least three subjects documented disease progression as increasing surface area of atrophic lesions over time, from the perifoveal area toward both midperiphery and the fovea, with a considerable enlargement over a 6- to 18-year period (Figure S2). Despite advanced disease stages and extensive areas of atrophy in some subjects, no individual exhibited an extinguished ERG, indicating relative preservation of peripheral retinal function. A large transitional zone between the most severely affected and healthy retina was commonly observed (Table S1). Importantly, all these characteristics were consistent across all individuals, regardless of their genotypes.

Approximately half of the affected individuals exhibited extraocular features of variable severity (Table 1). Some manifested disorders that are rather prevalent in the general population and were probably unrelated to their Mendelian condition, such as thyroid dysfunction, hypertension, type 2 diabetes, or ischemic stroke. Others, however, experienced symptoms that could be consistent with the spectrum of hereditary spastic paraplegia,

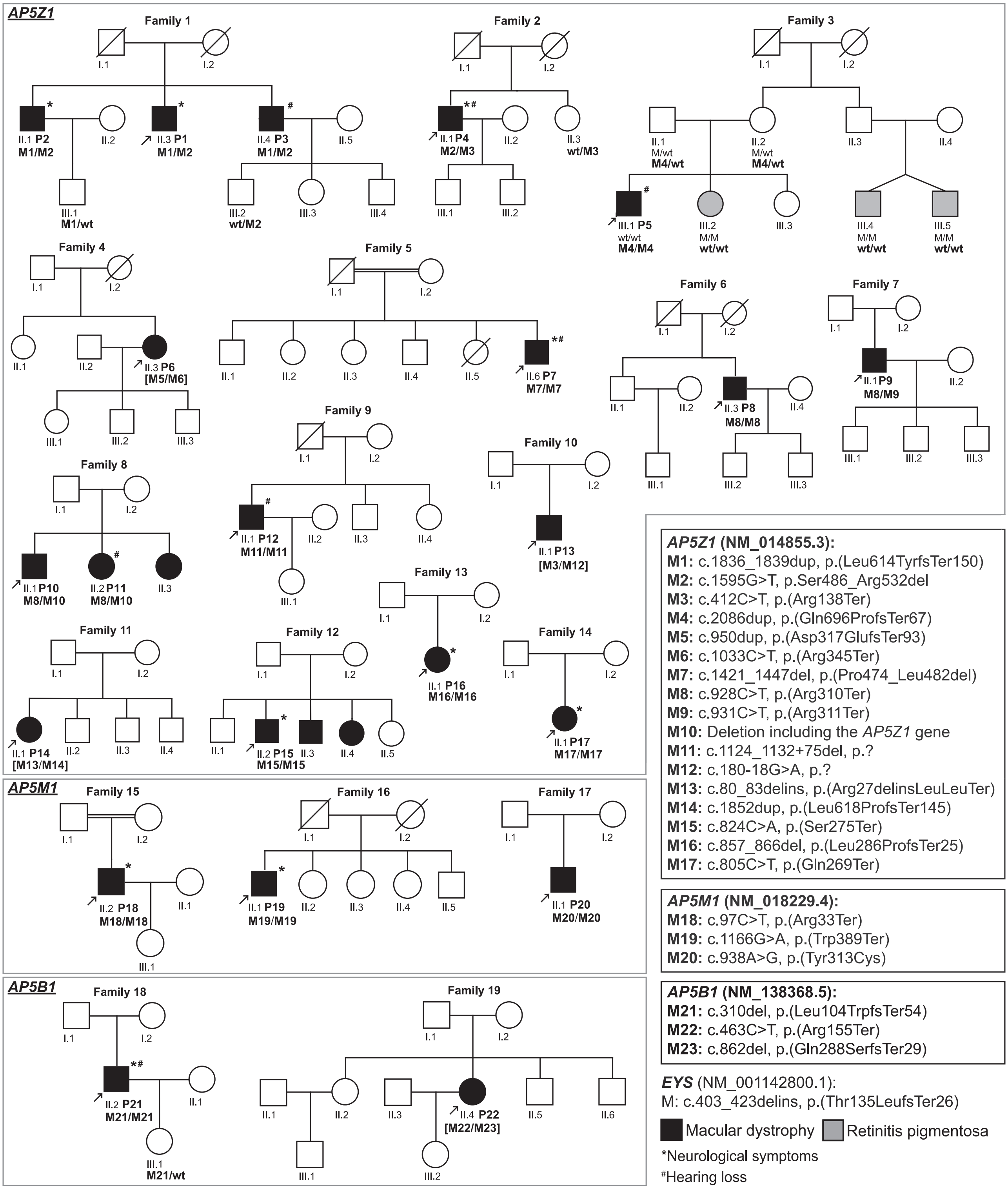

Figure 1. Pedigrees of the individuals analyzed

Disease-associated variants are indicated by MX; "wt" represents wild-type alleles. Arrows indicate probands, and square brackets indicate that the phase of the provided genotype is assumed but not proven by segregation analysis, due to lack of available biological material. The correct HGVS nomenclature for the variant in *AP5Z1* "c.80_83delins" is "c.80_83delinsTGCTGTAAACTGTAACTGTAAA" and for the variant in *EYS* "c.403_423delins" is "c.403_423delinsCTTTT."

although in most cases the phenotype was very mild and non-specific, including isolated pollakiuria, polyneuropathy, parkinsonism, or mild intellectual disability. For two individuals who presented with advanced neurological symptoms (P16 and P4, Table 1), brain magnetic resonance imaging (MRI) scans were conducted. MRI of P16

Table 1. Clinical and genetic data of individuals in the study

General Information						Clinical symptoms					Genotype			
Family ID	Affected individual ID	Referring center	Sex	Age at examination (years)	Age at onset (years)	Initial symptoms	IRD class	Main ocular features	Neurological symptoms	Other symptoms	Variant 1 (ID)	Variant 1	Variant 2 (ID)	Variant 2
AP5Z1 **(GenBank:** NM_014855.3**)**														
1	P1	Portugal (Lisbon)	M	63	50	central vision loss	MD	extensive central chorioretinal atrophy	pollakiuria	none reported	M1	c.1836_1839dup (p.Leu614 TyrfsTer150)	M2	c.1595G>T (p.Ser486_ Arg532del)
	P2	Portugal (Lisbon)	M	74	40	central vision loss	MD	extensive central chorioretinal atrophy	peripheral neuropathy with paraplegia	none reported	M1	c.1836_ 1839dup (p.Leu614 TyrfsTer150)	M2	c.1595G>T (p.Ser486_ Arg532del)
	P3	UK (London)	M	63	40	blurred vision	MD	extensive central chorioretinal atrophy	none	hearing loss (onset at 50 years old)	M1	c.1836_ 1839dup (p.Leu614 TyrfsTer150)	M2	c.1595G>T (p.Ser486_ Arg532del)
2	P4	Belgium (Ghent)	M	62	50	unilateral central vision loss, central scotoma, paresthesia	MD	extensive central chorioretinal atrophy	paresthesia in hands and feet, mild intellectual disability, ataxic gait	impression of hearing loss	M2	c.1595G>T (p.Ser486_ Arg532del)	M3	c.412C>T (p.Arg138Ter)
3	P5	Israel (Haifa)	M	43	43	reduced VA	MD	maculopathy with atrophy and deposits	none	hearing loss (onset at 40 years old)	M4	c.2086dup (p.Gln696 ProfsTer67)	M4	c.2086dup (p.Gln696Profs Ter67)
4	P6	Israel (Jerusalem)	F	55	53	reduced VA	MD	maculopathy with atrophy	none	none reported	M5	c.950dup (p.Asp317 GlufsTer93)	M6	c.1033C>T (p.Arg345Ter)
5	P7	UK (London)	M	56	47	central vision loss	MD	extensive central chorioretinal atrophy	pollakiuria	neurosensory hearing loss (onset at 35 years old), hypercholesterolemia, hypertension, asthma	M7	c.1421_ 1447del (p.Pro474_ Leu482del)	M7	c.1421_1447del (p.Pro474_ Leu482del)
6	P8	UK (Newcastle)	M	63	45	mostly asymptomatic	MD	extensive central chorioretinal atrophy	none	none reported	M8	c.928C>T (p.Arg310Ter)	M8	c.928C>T (p.Arg310Ter)
7	P9	UK (Liverpool)	M	63	50	central vision loss	MD	extensive central chorioretinal atrophy	none	hypertension, hearing loss (onset >50 years old)	M8	c.928C>T (p.Arg310Ter)	M9	c.931C>T (p.Arg311Ter)

(*Continued on next page*)

Table 1. *Continued*

General Information						Clinical symptoms					Genotype			
Family ID	Affected individual ID	Referring center	Sex	Age at examination (years)	Age at onset (years)	Initial symptoms	IRD class	Main ocular features	Neurological symptoms	Other symptoms	Variant 1 (ID)	Variant 1	Variant 2 (ID)	Variant 2
8	P10	UK (Liverpool)	M	65	45	central vision loss	MD	extensive central chorioretinal atrophy	none	none reported	M8	c.928C>T (p.Arg310Ter)	M10	deletion (7p22.1) including the AP5Z1 gene
	P11	UK (Liverpool)	F	63	late 40s	central vision loss	MD	extensive central chorioretinal atrophy	none	hiatus hernia, diverticulosis and colitis, hypertension, bilateral sensorineural hearing loss (onset in her late 50s)	M8	c.928C>T (p.Arg310Ter)	M10	deletion (7p22.1) including the AP5Z1 gene
9	P12	Spain (Valencia)	M	62	54	nyctalopia, reduced VA	MD	extensive central chorioretinal atrophy	none	impression of hearing loss in left ear, hypertension, type 2 diabetes	M11	c.1124_1132 + 75del (p.?)	M11	c.1124_1132 + 75del (p.?)
10	P13	Sweden (Lund)	M	59	40	problem with reading	MD	extensive central chorioretinal atrophy	none	none reported	M3	c.412C>T (p.Arg138Ter)	M12	c.180−18G>A (p.?)
11	P14	Germany (Tübingen)	F	32	31	metamorphopsias	MD	early maculopathy with deposits	none	thyroid dysfunction	M13	c.80_83delinsTGCTGTAAACTGTAACTGTAAA (p.Arg27delinsLeuLeuTer)	M14	c.1852dup (p.Leu618ProfsTer145)
12	P15	Germany (Tübingen)	M	53	44	poor central vision	MD	extensive central chorioretinal atrophy	parkinsonism	none reported	M15	c.824C>A (p.Ser275Ter)	M15	c.824C>A (p.Ser275Ter)
13	P16	Switzerland (Basel)	F	52	46	spastic atactic gait	MD	early maculopathy	spastic atactic paraparesis	none reported	M16	c.857_866del (p.Leu286ProfsTer25)	M16	c.857_866del (p.Leu286ProfsTer25)
14	P17	Spain (Madrid)	F	44	40	paresthesia	MD	early maculopathy with deposits	paresthesia in the right hand	none reported	M17	c.805C>T (p.Gln269Ter)	M17	c.805C>T (p.Gln269Ter)

(*Continued on next page*)

Table 1. *Continued*

General Information						Clinical symptoms					Genotype			
**Family ID**	**Affected individual ID**	**Referring center**	**Sex**	**Age at examination (years)**	**Age at onset (years)**	**Initial symptoms**	**IRD class**	**Main ocular features**	**Neurological symptoms**	**Other symptoms**	**Variant 1 (ID)**	**Variant 1**	**Variant 2 (ID)**	**Variant 2**
AP5M1 **(GenBank:** NM_018229.4**)**														
15	P18	Switzerland (Lausanne)	M	46	30	reduced central vision	MD	maculopathy with atrophy and deposits	mild intellectual disability	none reported	M18	c.97C>T (p.Arg33Ter)	M18	c.97C>T (p.Arg33Ter)
16	P19	Belgium (Ghent)	M	59	46	Parkinson symptoms	MD	extensive central chorioretinal atrophy	parkinsonism	type 2 diabetes, ischemic stroke	M19	c.1166G>A (p.Trp389Ter)	M19	c.1166G>A (p.Trp389Ter)
17	P20	Germany (Tübingen)	M	63	56	central vision loss	MD	extensive central chorioretinal atrophy	none	myocardial infarction, multiple allergies, chronic anal fistulas	M20	c.938A>G (p.Tyr313Cys)	M20	c.938A>G (p.Tyr313Cys)
AP5B1 **(GenBank:** NM_138368.5**)**														
18	P21	Greece (Heraklion)	M	68	55	reduced central vision, photophobia, nyctalopia	MD	extensive central chorioretinal atrophy	polyneuropathy	hearing loss (onset later in life)	M21	c.310del (p.Leu104 TrpfsTer54)	M21	c.310del (p.Leu104Trpfs Ter54)
19	P22	UK (London)	F	36	36	mostly asymptomatic	MD	early maculopathy with deposits	none	none reported	M22	c.463C>T (p.Arg155Ter)	M23	c.862del (p.Gln288Serfs Ter29)

M, male; F, female; IRD, inherited retinal degeneration; VA, visual acuity; MD, macular dystrophy.

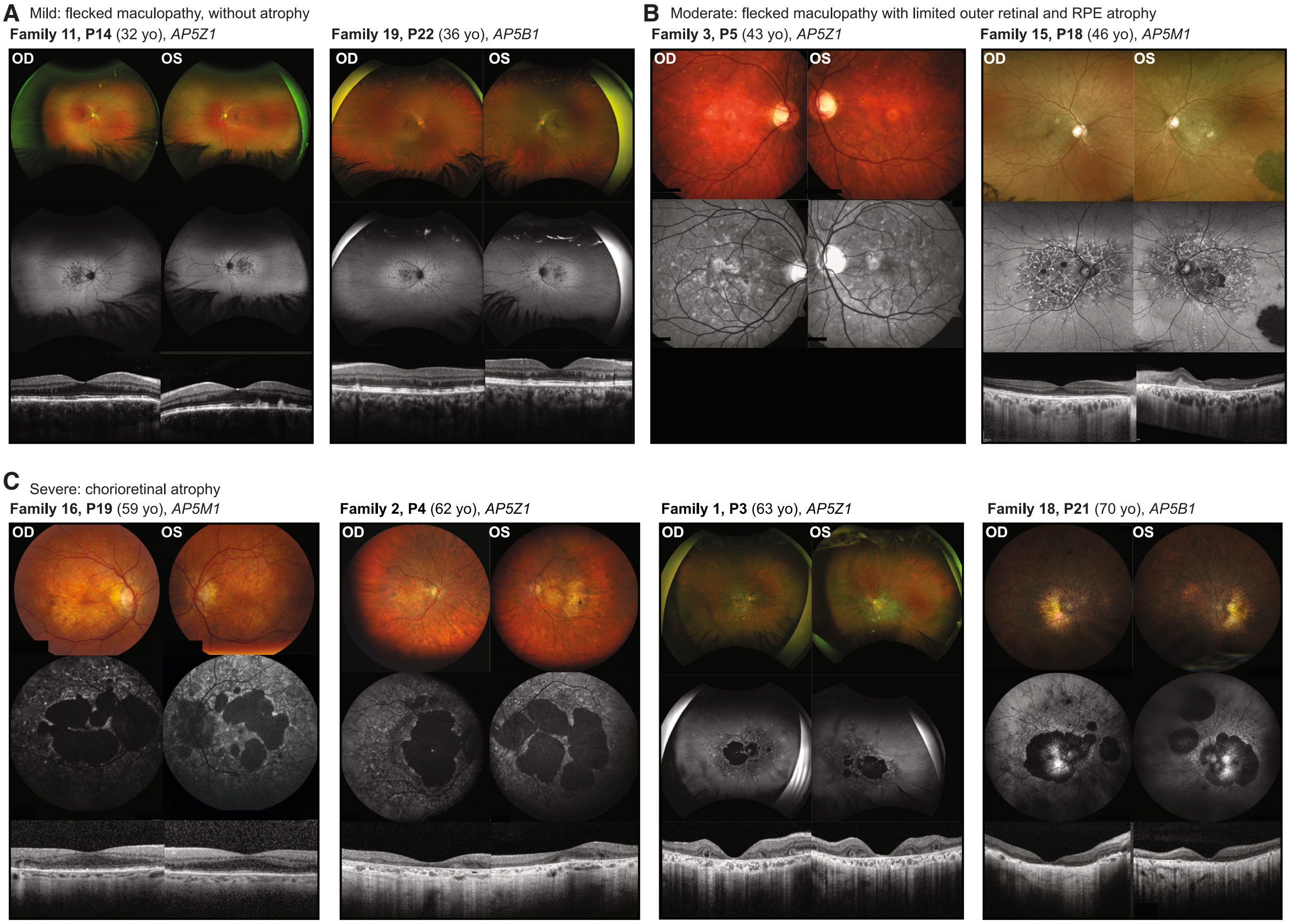

Figure 2. Multimodal retinal imaging of eight representative individuals from this study
In each panel, the top row shows color or pseudocolor fundus images, the middle row shows fundus autofluorescence images (FAF), and the bottom row shows optical coherence tomography (OCT) scans. OCT images were not available for individual P5 from family 3. Images are ordered according to the subjects' age. OD, right eye; OS, left eye; yo, years old.
(A) Two individuals (P14 with variants in *AP5Z1*, age 32; P22 with variants in *AP5B1*, age 36) with early-stage disease presenting with flecks without atrophy.
(B) Two individuals (P5 with variants in *AP5Z1*, age 43; P18 with variants in *AP5M1*, age 46) with limited atrophy of the RPE and outer retina and some deposits.
(C) Four individuals with advanced disease (P19 with variants in *AP5M1*, age 59; P4 with variants in *AP5Z1*, age 62; P3 with variants in *AP5Z1*, age 53; and P21 with variants in *AP5B1*, age 70) with variable degree of central chorioretinal atrophy.

(performed at the age of 49 years) revealed multiple small, round-shaped hyperintense lesions in the supratentorial, frontoparietal, and occipital white matter, as well as in the corpus callosum. In P4, the same test (performed at the age of 58 years) showed signs of generalized cortical and subcortical atrophy with discrete frontal chronic vascular leukoencephalopathy. Interestingly, six individuals experienced some degree of hearing impairment; which was confirmed not to be congenital, as it manifested later in life, between the 4th and 6th decade. Two additional subjects (P4 and P12) reported an impression of hearing loss (unilateral or bilateral), which was not confirmed by audiometry or other specialized tests (Table 1).

A degree of variability in extraocular features was also observed in individuals from the same family, hence carrying the same genotype in AP-5 genes. For instance, in family 1 the three affected brothers had similar retinal findings but different extraocular symptoms: P1 had pollakiuria, P2 peripheral neuropathy, and P3 hearing loss. In family 8, one of the affected siblings (P10) manifested non-syndromic retinal disease at his last examination (at age 65 years), while the other sibling (P11) developed non-specific extraocular features in her 5th and 6th decade of life, including bilateral sensorineural hearing loss.

Finally, since the disease manifests as a late-onset visual impairment, it is possible that younger individuals from these pedigrees, who were not examined or genotyped and are reported in Figure 1 as healthy, could develop this condition in the future.

Genetic analysis

All probands were negative for pathogenic variants in previously identified IRD-associated genes. New WES, new

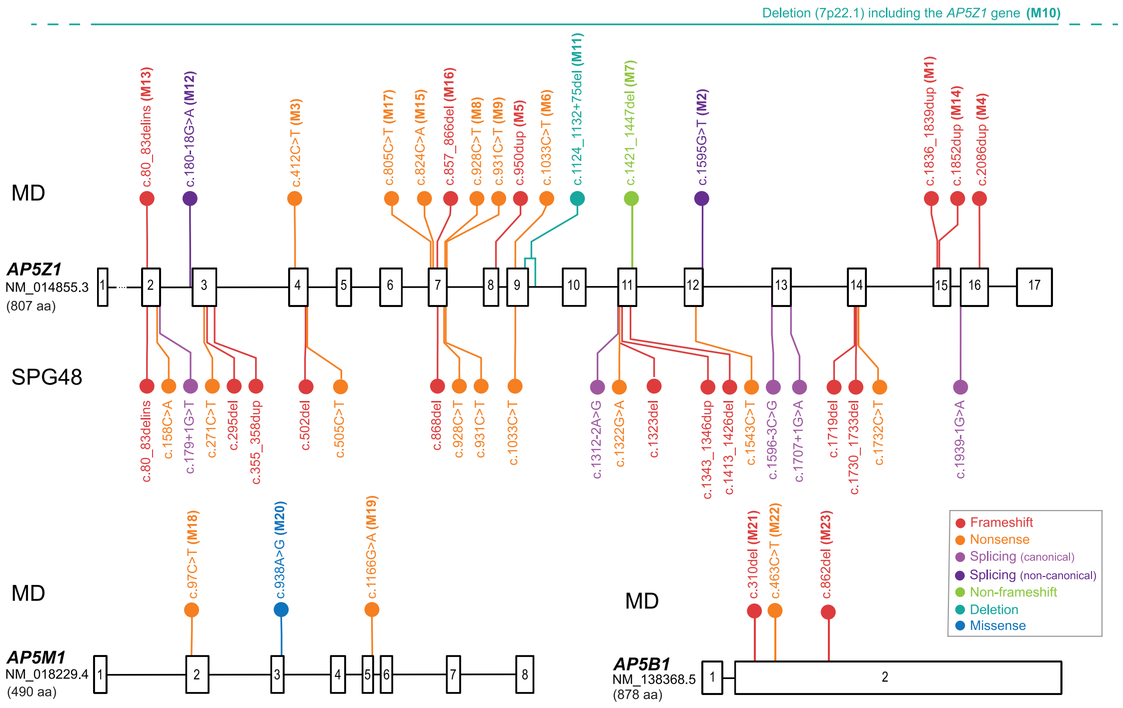

Figure 3. Schematic representation of the genetic variation spectrum of *AP5Z1*, *AP5M1*, and *AP5B1*, with respect to macular dystrophy and spastic paraplegia

The exons of each gene are indicated by numbered boxes, whereas introns are shown by horizontal lines. Their sizes with respect to actual genomic sequence, using canonical transcripts, are roughly proportional, although not to scale. 5′ UTRs and 3′ UTRs are omitted. The protein size, in number of amino acids (aa), is also indicated. Variants associated with an ocular phenotype (MD, macular dystrophy) and detected in this study (M1 to M23) are shown in various colors above the gene of interest, while variants previously reported in association with spastic paraplegia type 48 (SPG48) are indicated below the gene (only for *AP5Z1*). These latter DNA changes represent pathogenic or likely pathogenic variants reported in ClinVar as of July 2024, documented in the literature and/or linked to an affected individual. M10, a large deletion affecting the whole *AP5Z1* sequence, is indicated by a horizontal line. No clear genotype-phenotype correlation within or across conditions could be detected. Of note, all disease-causing variants identified are presumed loss-of-function alleles, with the exception of M20 in *AP5M1*, which is a missense (see text for details). The correct HGVS nomenclature for the variant in *AP5Z1* "c.80_83delins" is "c.80_83delinsTGCTGTAAACTGTAACTGTAAA."

WGS screening, or a reanalysis of previous sequencing data by using a customized analytical pipeline led to the identification of assortments of bi-allelic variants in *AP5Z1* (14 families), *AP5M1* (three families), and *AP5B1* (two families) (Table 1 and Figure 1). These three genes encode different subunits of the AP-5 complex, namely zeta, mu, and beta, respectively, and share structural similarity with corresponding subunits of other AP complexes.[25]

In total, we detected 23 distinct variants: 17 in *AP5Z1*, three in *AP5M1*, and three in *AP5B1* (Figure 3), the frequency of which in gnomAD[36] ranged from absent to 4.4×10^{-5}. Twenty-two out of these 23 changes were presumed loss-of-function (LoF) variants: nine nonsense, eight frameshift, one splice-site alteration, one partial and one full gene deletion, one non-frameshift deletion, and one single-nucleotide substitution creating a missense but predicted to alter pre-mRNA splicing and resulting in the ablation of an entire exon (M2, see below). The remaining DNA change was a missense (Tables 1 and S2). Most variants (17 out of 23) were not reported previously in ClinVar[48] or in the biomedical literature (Table S2).

We also estimated the putative prevalence of AP-5-related disorders by analyzing the frequency values of LoF alleles in the general population, as reported by gnomAD.[36] According to these data, 1 in 471,000 individuals should carry bi-allelic LoF variants in *AP5Z1*, making *AP5Z1*-associated disease the most prevalent one. *AP5B1*-related disease would have a prevalence of 1 in 5.9 million, and disorders linked to *AP5M1* variants would be present in 1 in 6.5 million people. If deleterious variants in *AP5S1* were also associated with a pathological phenotype, the prevalence of such disease would be rather rare, i.e., 1 in 105 million. Overall, the combined prevalence across all AP-5-related conditions was estimated at 1 in 407,000 people, translating to approximately 20,000 potentially affected individuals in the world—comprising 17,400 *AP5Z1* cases, 1,400 *AP5B1* cases, 1,200 *AP5M1* cases, and only 80 *AP5S1* cases. As a reference, late-onset MDs have a collective prevalence of roughly 1 in 10,000 people, as ascertained by integrating existing data on individuals with *ABCA4* (late-onset Stargardt disease, MIM: 248200), *PRPH2* (central areolar choroidal dystrophy, MIM: 613105), and *BEST1* (late-onset MD, MIM: 153700) pathogenic variants.[49–53]

Variants in *AP5Z1*

Family 1 was from Portugal and included three affected individuals (P1, P2, and P3), born to healthy parents. The proband, P1, was found to carry two heterozygous variants in *AP5Z1*: c.1836_1839dup (p.Leu614TyrfsTer150) (M1, reference sequences for *AP5Z1* mRNA and protein: GenBank: NM_014855.3 and NP_055670.1, respectively) and c.1595G>T (p.Ser486_Arg532del) (M2, an SNV leading to aberrant splicing; see below). Molecular analysis of his two affected brothers and his two unaffected nephews confirmed these variants to be in *trans* and to co-segregate with the disease in a recessive fashion (Figure 1). Proband P4 from family 2 was a simplex case with MD from Belgium, born to unaffected parents and with unaffected offspring. He was also found to carry M2, in a compound heterozygous state with the nonsense variant c.412C>T (p.Arg138Ter), or M3. Co-segregation analysis confirmed the bi-allelic nature of his genotype (Figure 1).

In family 3, an Israeli pedigree of North African Jewish descent, the proband (P5) presented with MD and late-onset hearing loss. He carried a homozygous frameshift variant (c.2086dup [p.Gln696ProfsTer67], M4). Intriguingly, he had three relatives with a different retinal disease, RP, who did not share the *AP5Z1* genotype. Molecular analysis confirmed that these individuals had a different etiology for their disorder, a homozygous known pathogenic frameshift variant in *EYS* (c.403_423delinsCTTTT [GenBank: NM_001142800.1] [p.Thr135LeufsTer26], ClinVar: 812320). This latter variant, previously identified as a founder mutation for autosomal recessive RP (MIM: 602772) in the Israeli Moroccan Jewish subpopulation,[54] aligned with the family's origin. All variants segregated with the respective diseases within the family and were confirmed to be inherited from the unaffected carrier parents (Figure 1). Individual P6 from family 4, a pedigree of Ashkenazi Jewish origin, was also from Israel. She was a simplex case with MD, born to unaffected parents and with three unaffected children. She was found to carry two LoF variants in *AP5Z1*, c.950dup (p.Asp317GlufsTer93) (M5) and c.1033C>T (p.Arg345Ter) (M6). Co-segregation analysis was not possible, due to the lack of biological material from family members, but gnomAD reported these two alleles to belong to two different haplotypes (100% estimated likelihood, with no individuals carrying both variants), strongly supporting the bi-allelic nature of the detected genotype.

Families 5, 6, 7, and 8 were all from the United Kingdom. Family 5 was a consanguineous pedigree of Pakistani descent. The proband, P7, presented with MD and late-onset hearing loss (Table 1). Molecular analysis revealed a homozygous non-frameshift deletion in *AP5Z1* (c.1421_1447del [p.Pro474_Leu482del], or M7), involving the ablation of 9 amino acid residues highly conserved across vertebrates. He was also positive for a rare homozygous missense (c.22G>C [GenBank: NM_173477.5] [p.Ala8Pro]), classified as a variant of uncertain significance (VUS) in ClinVar: 1687494, in *USH1G*, a gene linked to Usher syndrome type I (MIM: 606943). This variant is unlikely to be contributing to the retinopathy observed in this individual because *USH1G* is associated with a different condition, characterized by congenital profound hearing loss and early-onset RP.[55] Of note, this individual had significant homozygous regions across the genome (more than 700 Mb,[56] overall, for which more than 20% of all genomic variants appear in the homozygous state). Families 6, 7, and 8 included individuals of British ancestry, again all presenting with MD. P8, the proband of family 6, was homozygous for the nonsense variant c.928C>T (p.Arg310Ter), or M8. The same DNA change was present in P9, from family 7, as well as in P10 and P11, two siblings from family 8, in a compound heterozygous state with two other variants (Figure 1). Based on the visualization of sequencing data,[37] in P9 M8 was detected in *trans* with another nearby nonsense variant, c.931C>T (p.Arg311Ter) (M9), whereas in P10 and P11 it was in *trans* with a large deletion (M10) on chromosome 7p22.1, which included the full sequences of *AP5Z1*, *RADIL*, and *PAPOLB*, as well as parts of the genes *FOXK1* and *MMD2* (g.4808369_4950979del [GenBank: NC_000007.13]).

Subject P12 from family 9 was a simplex case from Spain, with signs and symptoms consistent with MD. He was born to unaffected parents and had an unaffected daughter and two unaffected siblings (Figure 1). Molecular analysis revealed the presence of another deletion in *AP5Z1*, in a homozygous state, involving the end of exon 9 and part of intron 9 (c.1124_1132+75del [p.?], or M11). *In silico* tools predicted this DNA change to result in the skipping of the full exon and a shift of the reading frame (p.Ala323GlyfsTer52) (Table S2).

Families 10, 11, 12, 13, and 14 were from Sweden, Germany, Germany (of Turkish ancestry), Switzerland, and Spain, respectively, and were all ascertained as pedigrees composed of affected individuals with unaffected parents who did not provide biological material for further genetic testing. All subjects were positive for pathogenic variants in *AP5Z1* (Figure 1). Individual P13 from family 10 was found to carry two heterozygous variants, c.412C>T (p.Arg138Ter) (M3), mentioned previously, and c.180−18G>A (p.?) (M12). This latter change was predicted by SpliceAI[57] to alter canonical splicing by creating a new strong acceptor site, in turn leading to the shift of the reading frame and a premature stop codon (p.Arg60HisfsTer41) (Table S2). Although co-segregation analysis was not possible, data from gnomAD indicated that the M3 and M12 changes are likely to be in *trans*, as they occur individually in presumably different subjects. Two heterozygous variants, c.80_83delinsTGCTGTAAACTGTAACTGTAAA (p.Arg27delinsLeuLeuTer) (M13) and c.1852dup (p.Leu618ProfsTer145) (M14), were detected in P14 from family 11. Again, due to the absence of DNA from parents or unaffected siblings, we could not prove the bi-allelic nature of these LoF variants. However, M13 was detected previously in two French siblings with spastic paraplegia,[58] suggesting that it belongs to a

different haplotype with respect to M14. Probands from Families 12, 13, and 14 were all homozygotes for LoF alleles: c.824C>A (p.Ser275Ter) (M15), c.857_866del (p.Leu286ProfsTer25) (M16), and c.805C>T (p.Gln269 Ter) (M17), respectively.

Variants in *AP5M1*

Probands from three additional pedigrees, families 15, 16, and 17, presented with ophthalmic features similar to those displayed by individuals with pathogenic variants in *AP5Z1*, described above (Table 1). All of them were simplex cases, born to unaffected parents (Figure 1). P18, from family 15, was a male of Portuguese origin from Switzerland. His parents were genetically related. Molecular analysis revealed the presence of a homozygous nonsense variant in *AP5M1*, c.97C>T (p.Arg33Ter), or M18 (reference sequences for mRNA and protein: GenBank: NM_018229.4 and NP_060699.3, respectively). P19 from family 16 was from Belgium and was found to be homozygous for another nonsense DNA change, c.1166G>A (p.Trp389Ter), or M19. Finally, P20 from family 17, an individual of Turkish ancestry ascertained in Germany, carried a homozygous missense variant, c.938A>G (p.Tyr313Cys) (M20). This DNA change affects an amino acid residue that is conserved across all vertebrates, as well as all AP5M1 human paralogs (AP1M1, AP2M1, AP3M1, and AP4M1; Figure S3), had a MutScore value of 0.924 (maximum = 1.000)[59] and was predicted to be deleterious by Franklin (https://franklin.genoox.com), with an aggregated *in silico* score from multiple tools of 0.84 (maximum = 1.00). Notably, M20 was the only true missense change among all of the causative variants detected in this study and, interestingly, a missense affecting Tyr284 of AP4M1, the corresponding amino acid residue of Tyr313 in AP5M1, is linked to spastic paraplegia type 50 (MIM: 612936).[60]

Variants in *AP5B1*

Individual P21 belongs to family 18, from Greece, while individual P22 was from a pedigree of Afghani origin from the United Kingdom (family 19). Both probands were simplex cases, presenting with signs of MD (Figure 1). They both carried variants in *AP5B1*, encoding a third component of the AP-5 complex. P21 was found to be homozygous for the LoF change c.310del (p.Leu104TrpfsTer54), or M21 (GenBank: NM_138368.5 and NP_612377.4, respectively), whereas P22 was a likely compound heterozygote for two other LoF variants, c.463C>T (p.Arg155Ter), or M22, and c.862del (p.Gln288SerfsTer29), or M23. The bi-allelic nature of these variants in P22 was very likely, since gnomAD reported the presence of M22 independently from M23 in two heterozygous Asian individuals, but this could not be proven experimentally owing to the non-availability of additional family members. Even though the three variants described above are located in the last exon of *AP5B1* (Figure 3), they all remove more than two-thirds of the protein's full sequence and are therefore extremely likely to represent full LoF alleles.

Functional validation of M2

The M2 change, c.1595G>T (p.Ser486_Arg532del), occurring in the last base of exon 12 of *AP5Z1*, was the second most frequent variant detected in our cohort. Although this base substitution would theoretically result in the missense p.Arg532Met, it was predicted to affect pre-mRNA splicing by four *in silico* tools, SpliceAI[57] (score: Δ0.76 out of 1.00, donor loss), dbSNV[61] (0.99 out of 1.00), SQUIRLS[62] (0.99 out of 1.00), and MaxEntScan[63] (Δ5.72, strong),[64] and therefore likely to result in an LoF allele. To validate these predictions, we analyzed the RNA (cDNA) of leukocytes from individual P1 (carrying M2 heterozygously) and a control individual, as well as the RNA from a healthy human retina sample. The analysis revealed that M2 leads to an aberrant splicing event resulting in the skipping of exon 12 of *AP5Z1* and causing an in-frame deletion of 47 amino acid residues, r.1455_1593del (p.Ser486_Arg532del). This aberrant transcript was absent in both the control sample and in cDNA from the retina (Figure 4), allowing us to classify M2 as a likely pathogenic splice-altering variant (Table S2).[34]

Localization of AP5Z1, AP5M1, and AP5B1 in the human RPE and in iPSC-RPE monolayers

Previous immunofluorescence microscopy in fibroblasts derived from individuals with spastic paraplegia type 48,[65] as well as studies on cellular[25] and animal[66] models of *AP5Z1* knockdown, highlighted the importance of the AP-5 complex in tissues with high endolysosomal activity. We therefore reasoned that, for a retinal phenotype, primary defects linked to AP-5 deficiency would likely occur in the RPE, a tissue for which intracellular vesicular activity is elevated. Such activity is needed to maintain cellular homeostasis as well as the continuous phagocytosis of fragments of photoreceptors' outer segments. We thus assessed the presence and localization of the three components of the AP-5 complex studied here by immunofluorescence on human RPE flat-mount sections. Confocal microscopy imaging revealed punctate labeling of AP5Z1, AP5M1, and AP5B1 in the cytoplasm of RPE cells, with the highest abundance for AP5B1 and the lowest for AP5M1 (within the limits of protein quantification by immunofluorescence) (Figure 5).

We then used human iPSC-RPE monolayers to further investigate the localization of these AP-5 components with respect to markers of distinct cellular compartments involved in the endolysosomal trafficking as well as their various vesicular maturation stages (Figure S4A). Immunofluorescence imaging of those sections confirmed the punctate staining patterns of AP5Z1, AP5M1, and AP5B1, revealing their strongest co-localization with Rab7, a marker of late endosomes, and partial overlap with TGN46-positive Golgi-derived vesicles (Figures S4B and S6). Conversely, no significant co-localization was detected

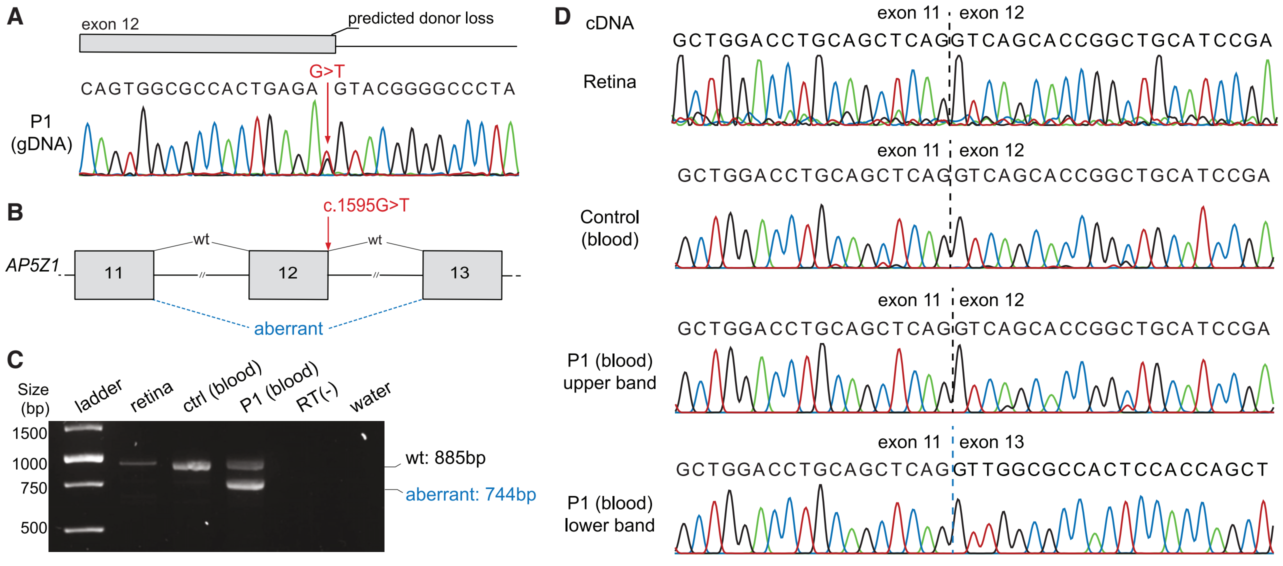

Figure 4. Effect of the M2 variant in *AP5Z1* on pre-mRNA splicing
(A) Schematic representation of the last bases of exon 12 and first bases of intron 12 of *AP5Z1*, corresponding to the electropherogram of the PCR product obtained from the genomic DNA (gDNA) of individual P1. The arrow indicates the position of the variant M2, a single-nucleotide change of guanine into thymine (G>T).
(B) Diagram of exons 11–13 of *AP5Z1*, including the position of the M2 variant and the aberrant splicing event detected. The canonical mRNA transcript is indicated by "wt."
(C) Electrophoresis of RT-PCR products from healthy retina, leukocytes of a control individual (ctrl), and leukocytes of the affected individual P1, carrying M2 heterozygously, *in trans* with M1. The amplification product of the canonical transcript measures 885 bp in length, while the aberrant one has a size of 744 bp, corresponding to the skipping of the whole exon 12. RT(−), non-reverse transcriptase control reaction; water, negative control of amplification.
(D) Electropherograms representing the PCR products shown in (C). The retina and the control sample contain only the canonical transcript, while P1 presents both wt (corresponding to the upper band on the electrophoresis gel) and aberrant (corresponding to the lower band) transcripts.

with LAMP2 (lysosomal marker), EEA1 (early endosomal marker), or TfR (a marker of recycling endosomes) (Figure S4B). High-resolution imaging provided additional insights into the subcellular distribution of these AP-5 proteins, predominantly observed on the apical side of the iPSC-RPE monolayer, with no overlap with the nuclear region, as shown in cross-sectional views (Figure 6). In particular, within the apical cytoplasm, they seemed to be organized in tubular structures, potentially extending through the microvilli region, although the functional significance of this arrangement *in vivo* remains unclear (Figure S5 and Video S1).

These results were consistent with previous reports investigating the presence of active AP-5 complexes in other cells,[67,68] supporting the hypothesis that AP-5 has a key role in the maintenance of correct lysosomal pathways by facilitating the sorting step between late endosomes and the *trans*-Golgi network, also in the RPE.

Discussion

In this study, we investigated 22 individuals affected with recessive MD from 19 unrelated families, mostly of European descent. All study subjects exhibited a consistent retinal phenotype, with the median age of symptom onset being in their 5th decade. Based on the available clinical data, the disease seems to include flecks at an early stage, incomplete retinal atrophy at an intermediate stage, and complete chorioretinal atrophy extending from the macula toward the periphery at later stages. This pattern was observed in all individuals, regardless of their genotypes, and was supported by longitudinal data from at least three subjects. Fleck-like deposits are common at early and intermediate stages of IRDs with RPE involvement, such as Stargardt disease, Stargardt-like diseases, pattern dystrophies, and central areolar choroidal dystrophy.[2,69] Features distinguishing ocular findings in this study from *ABCA4*-associated retinopathy are foveal sparing, early peripapillary involvement, and reticular pigment clumping in the peripheral retina.

All affected individuals carried variants in any of three genes encoding subunits of the AP-5 complex: *AP5Z1* (previously associated with spastic paraplegia type 48, SPG48), *AP5M1*, and *AP5B1*. Specifically, we identified 23 unique variants, most of which (74%) were not described previously. Overall, the distribution of these pathogenic variants across the different AP-5 genes aligned well with the relative prevalence of potentially pathogenic bi-allelic changes in the general population. *AP5Z1* cases are predicted to be the most common, followed by pathogenic genotypes in *AP5B1* and *AP5M1*. While it is still unclear whether deleterious variants in *AP5S1* are associated with disease, population-based estimates suggest that such

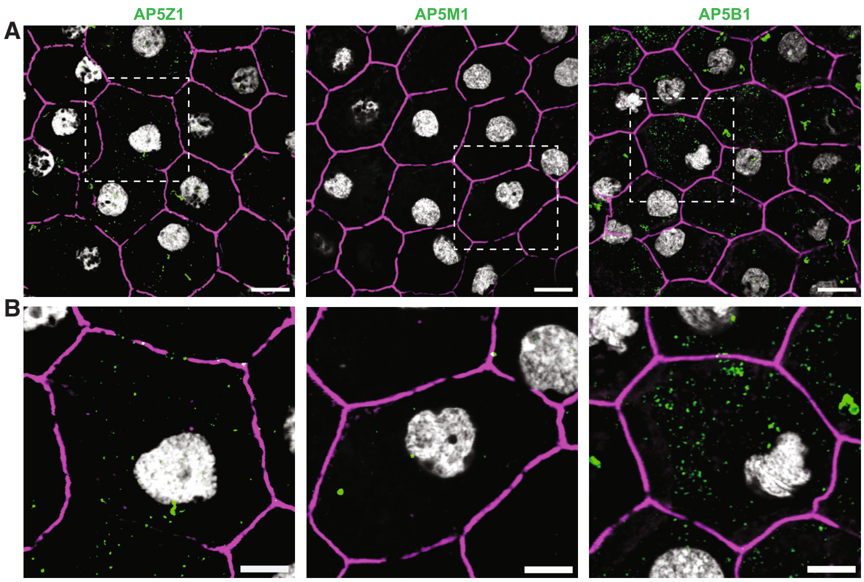

Figure 5. AP5Z1, AP5M1, and AP5B1 display punctate localization in human RPE explants
(A) En-face imaging of flat-mount sections is presented, showing the localization of AP5Z1, AP5M1, and AP5B1 (green), in the cytoplasm of RPE cells. Phalloidin (magenta) stains actin filaments, revealing individual cells' plasma membranes, which are arranged in a typical hexagonal mosaic pattern within this tissue. DAPI (4,6-diamidino-2-phenylindole; gray) stains nuclei. Scale bars, 10 μm.
(B) Magnified images of selected regions, focusing on individual RPE cells, demonstrating the punctate labeling of AP5Z1, AP5M1, and AP5B1, respectively. Scale bars, 5 μm.

events would be extremely rare. Interestingly, most of the DNA changes detected (22 out of 23) were predicted to be LoF variants, supporting their pathogenic role and clearly indicating that the retinal phenotype observed clinically is a consequence of the lack of AP-5 components at the cellular level. Interestingly, one of these variants (M2), expected to result in a single amino acid substitution, was demonstrated through a functional assay to cause the skipping of the entire exon 12 of *AP5Z1* and was classified as a splice-disrupting change. Like for M7, M2 resulted in the in-frame deletion of several amino acid residues of AP5Z1, in a region that is phylogenetically conserved. Moreover, most variants were detected only in one family, highlighting the allelic heterogeneity and rarity of this AP-5-related class of IRDs. Some DNA changes (M2, M3, and M8) seemed to have occurred early in the history of the European population and be relatively prevalent in this broad continental group, as they mostly occurred in a compound heterozygous state in families from Portugal, Belgium, Sweden, and the United Kingdom. Altogether, these data suggest common underlying disease mechanisms and, potentially, shared therapeutic approaches.

In the context of SPG48 due to variants in *AP5Z1*, visual impairment has been noted as a non-specific clinical feature of the disease spectrum, albeit only in five syndromic individuals from a single study and from a review paper, out of all the cases described so far.[65,70] These subjects primarily exhibited spastic paraparesis, sensory and motor neuropathy, ataxia, dystonia, myoclonus, and parkinsonism. While the brief ophthalmic description provided did not clearly associate defects in this gene with IRDs, the only fundus image available from one of these subjects closely resembles the chorioretinal atrophy observed in all the individuals from our study with advanced disease.[70] Ocular abnormalities, such as pigmentary retinal degeneration, ophthalmoplegia, optic atrophy, cataracts, and nystagmus, are common in other forms of spastic paraplegia, such as SPG7 (MIM: 607259), SPG35 (MIM: 612319), or SPG45 (MIM: 613162), not linked to *AP5Z1*, where ophthalmic changes may precede extra-ocular motor symptoms.[71] Conversely, *AP5M1* and *AP5B1* were not previously associated with any human hereditary disease.

In our cohort, all affected individuals exhibited a consistent ophthalmic phenotype characterized by progressive MD, as described above, with a variable range and severity of extraocular symptoms. A subset of them had comorbidities that are relatively prevalent in the general population and are probably unrelated to the defects in the AP-5 genes detected in their genome. However, ten individuals presented neurological features that could be compatible with the disease spectrum of spastic paraplegia. Interestingly, eight subjects experienced some degree of late-onset hearing impairment (either confirmed sensorineural loss or a subjective feeling of hearing deterioration), which aligns with sporadic reports of hearing impairment in individuals with SPG48.[65,72] Importantly, however, most affected individuals did not report significant extraocular symptoms (not even at advanced ages), suggesting that variants in *AP5Z1*, *AP5M1*, and *AP5B1*, including bi-allelic LoFs likely resulting in no protein products, mostly cause a non-syndromic IRD phenotype. Indeed, it is not uncommon for LSDs to manifest as an isolated retinal condition, which can be variant specific (e.g., for mutations in *HGSNAT*[73]) or variant-independent (e.g., *CLN3*-associated diseases, where the same DNA change can cause syndromic or non-syndromic phenotypes).[74] Our analysis did not reveal any clear genotype-phenotype correlations between the specific AP-5 genes bearing pathogenic variants, their alternative transcripts, and the range and severity of symptoms manifested by affected individuals. The same was true for a possible relationship between the position of a given variant with respect to the protein primary sequence and the ascertained phenotypes, also considering known SPG48 pathogenic variants in AP5Z1.

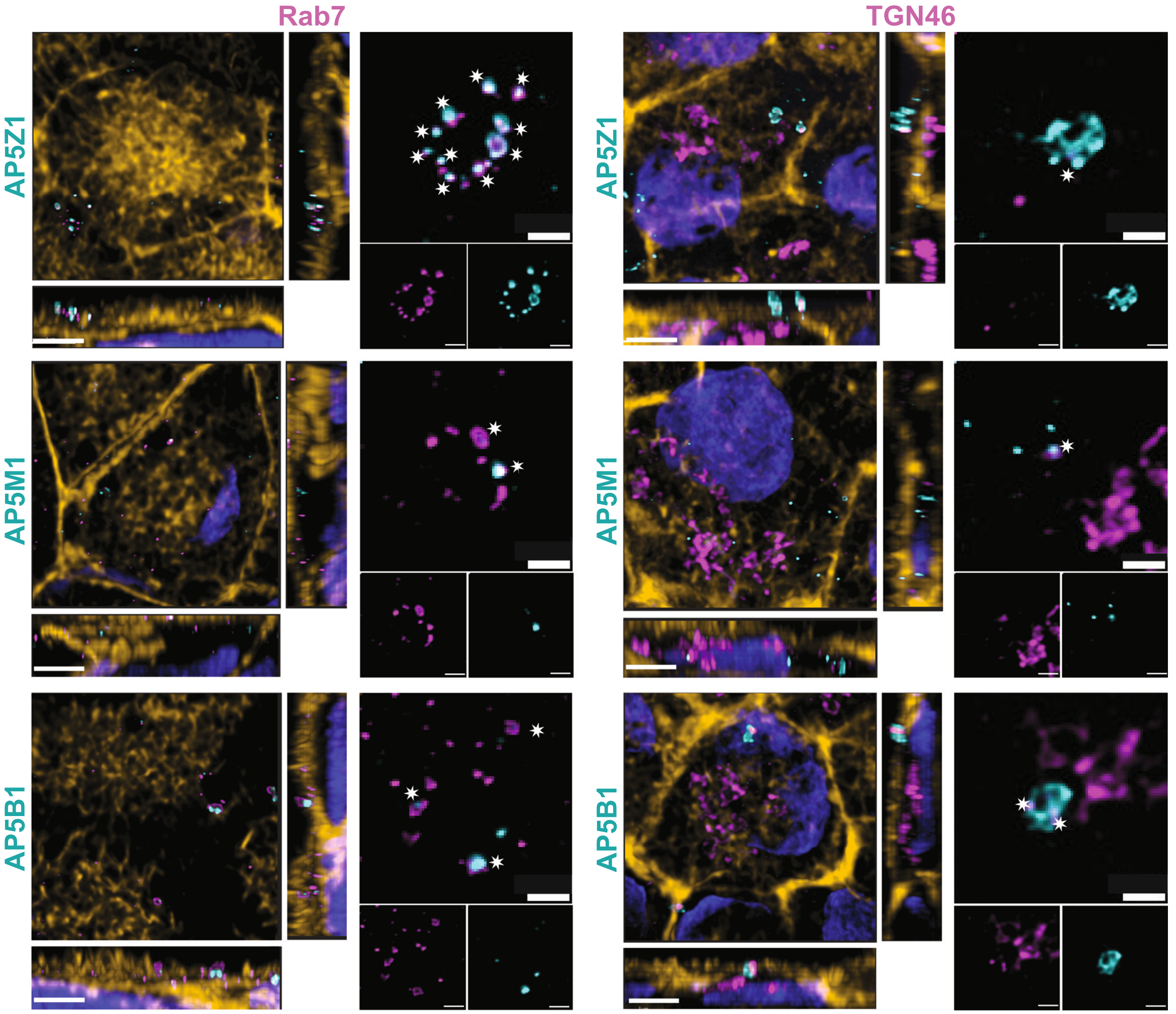

Figure 6. AP5Z1, AP5M1, and AP5B1 co-localize with markers of late endosomes and the *trans*-Golgi network in human iPSC-RPE cell lines
Immunofluorescence of an iPSC-derived RPE monolayer shows co-staining of all AP-5 components studied here (cyan) with Rab7 (magenta), a marker of late endosomes (left) or TGN46 (magenta), a marker of the *trans*-Golgi network structures (right side of each panel). White asterisks on higher-magnification images highlight regions of co-localization, revealed either by white areas (indicative of magenta and cyan overlap) or by magenta areas engulfing cyan spots. En-face and cross-sectional views of the same images are shown on the left of each panel. Phalloidin (yellow) stains actin filaments and plasma membranes, and DAPI (blue) stains nuclei. Scale bars, 3 μm (images with phalloidin staining) and 1 μm (images without phalloidin staining).

M6 (previously associated with SPG48[70]) in our cohort led to non-syndromic ocular disease in individual P6. Similarly, M8, associated in ClinVar with SPG48, was identified in three unrelated families from our study, with no subjects displaying manifest neurological symptoms. In particular, one of the probands, P8, carried it homozygously and yet displayed non-syndromic retinal deficit, which was confirmed by a normal MRI of the brain and the spinal cord, performed at the age of 63 years. It is possible, however, that the limited number of considered cases does not allow any statistically meaningful analysis and that further studies are needed to unambiguously prove or refute any notable genotype-phenotype relationship.

In humans, there are five adaptor protein complexes (AP-1 to AP-5), all involved in distinct pathways of intracellular vesicular trafficking. In general, they are involved in the formation of vesicles that transport cargo proteins to various cellular compartments, such as the *trans*-Golgi network, endosomes, lysosomes, and the plasma membrane.[75–77] Mutations in components of all five AP complexes have been found to have consequences for human health, leading to the so-called adaptinopathies, a class of neurodevelopmental and neurometabolic disorders of membrane trafficking.[25,78] While this term provides a relatively broad clinical and molecular definition, specific studies of the AP-5 complex have shown that its depletion

results in the accumulation of aberrant endolysosomes, which exhibit multi-lamellar structures filled with storage material similar to those observed in LSDs.[21] AP-5 deficiency also impairs the retrieval of several proteins from late endosomes back to the Golgi apparatus, suggesting that AP-5 facilitates a late-acting retrieval pathway that contributes to normal lysosomal homeostasis.[21] Furthermore, the absence of functional AP-5 leads to a notable increase in the size and number of LAMP1-positive puncta (revealing late endosomes, lysosomes, and endolysosomes), in turn indicating lysosomal dysfunction and suggesting a specific role for the AP-5 complex in the recycling of lysosomal enzymes and membrane proteins.[25] Interestingly, the loss of AP5Z1 and AP5B1 causes a concomitant reduction in levels of AP5M1, likely due to a decreased structural stability of the resulting aberrant AP-5 complexes.[23,25] Additionally, depletion of either AP5B1 or AP5M1 causes the relocalization of lysosomal membrane and retromer proteins as well as an increase in the size of multivesicular bodies near the Golgi apparatus.[23] Taken together, these observations suggest that the AP-5 complex is involved in late endosomal sorting and in lysosomal maintenance and that its disruption impairs lysosomal function and endosome trafficking, leading to a previously unrecognized type of LSD.[25] The AP-5 complex also interacts with spatacsin (SPG11, gene: *SPG11*, MIM: 610844) and spastizin (SPG15, gene: *ZFYVE26*, MIM: 612012), two proteins involved in the endosomal-lysosomal pathway.[68] These proteins are in turn associated with spastic paraplegia types 11 (MIM: 604360) and 15 (MIM: 270700), respectively, two conditions that include retinal phenotypes bearing similarities with those observed in the subjects of our study.[71,79] In particular, the mild to moderate flecked maculopathy with limited atrophy, predominantly observed in younger individuals, closely resembled ocular features of Kjellin syndrome, a late-onset neuro-ophthalmologic disorder caused by pathogenic variants in *SPG11* and *ZFYVE26*.[80–82] Previous studies have also shown that fibroblasts derived from individuals carrying mutations in *SPG11* and *ZFYVE26*, as well as RPE cell lines depleted for these genes, exhibit significantly enlarged lysosomes and endolysosomes in addition to an accumulation of autolysosomes.[83,84] These data strongly suggest that the retinal abnormalities observed in individuals carrying AP-5 variants could be the consequence of RPE dysfunction, as is the case in many other IRD types.[85–89]

The RPE monolayer, located dorsally to the neuroretina, is crucial for maintaining retinal health and function. One of its primary roles is the daily phagocytosis of fragments of photoreceptor outer segments (POSs), where lysosomes facilitate the degradation of such internalized structures within fused phagolysosomes. Its impairment in the RPE results in the buildup of harmful aggregates that lead to damage of the outer retina, a process observed in conditions such as Stargardt disease and choroideremia,[90] as well as in LSD caused by *CLN3* pathogenic variants, where patient-derived iPSC-RPE lines exhibit disrupted POS phagocytosis.[20] In addition to heterophagy (breakdown of extracellular material), the RPE also relies heavily on autophagy, which involves the formation of autophagosomes that fuse with lysosomes to degrade damaged cellular components. This is vital for maintaining cellular homeostasis and is regulated by various signaling pathways, which influence lysosomal biogenesis and function.[91,92] A previous study using animal models suggested that the AP-5 complex can also play a significant role in regulating the autophagic flux and that its impairment leads to the accumulation of undegraded autophagic cargoes, contributing to cellular stress and degeneration.[66] Recent research has also underscored the importance of maintaining correct autophagic flux and lysosomal integrity in the RPE to prevent retinal degeneration.[92,93] Therapeutic strategies targeting these pathways, such as enhancement of degradative enzyme activity or activation of the AMPK pathway, have demonstrated potential in restoring lysosomal clearance and protecting the RPE from degeneration in the context of Stargardt disease.[94,95] Additionally, the rescue of lysosomal function by several lysosome-enhancing compounds has emerged as a promising therapeutic strategy for treating LSDs, including SPG15.[96] These data further emphasize the critical role of lysosomes in the proper functioning of the RPE and open the possibility of developing treatments for AP-5-associated disorders.

To the best of our knowledge, the localization and role of the AP-5 complex in retinal tissues have not been studied previously. Our data on human RPE explants and wild-type iPSC-derived RPE monolayers show punctate labeling of all three AP5Z1, AP5M1, and AP5B1 proteins on the apical side of the cell. Furthermore, we demonstrate co-localization of these AP-5 subunits with markers of late endosomes and the *trans*-Golgi network, suggesting that the AP-5 complex is primarily associated with these compartments in RPE cells, consistent with findings from other cellular models.[68,97] Interestingly, we did not observe significant co-staining of any of the studied AP-5 proteins with LAMP2, a lysosomal marker, whereas prior studies have shown partial localization of AP5Z1 to lysosomes.[23,68] This may be due to differences in the studied cell types, variations in experimental conditions, or the dynamic nature of vesicular trafficking, highlighting the need for further exploration of the roles of the AP-5 complex in these intracellular processes. Although we clearly reveal the presence of the AP-5 complex in the RPE, supporting the potential role of this tissue in early events of pathogenesis for the disorder described, in this work we do not demonstrate any direct link between this tissue and macular degeneration. Further experimental research, building on the genetic findings presented here, is needed to establish the precise molecular etiology of AP-5-associated vision loss.

In summary, through a large multi-national collaborative effort involving multiple centers, we have identified pathogenic variants in the genes *AP5Z1*, *AP5M1*, and *AP5B1* as an independent cause of a recessive form of

retinal disease characterized by the progressive loss of macular photoreceptors. This clinical phenotype likely results from defects of the AP-5 complex as a whole at the cellular level, thus broadening the spectrum of previously described adaptinopathies. It is plausible that future studies will identify causative variants in the fourth component of the AP-5 complex, *AP5S1*, and associate it with a similar human phenotype. Due to the particular clinical and molecular features observed, we propose designating this phenotype as a distinct clinical subtype of IRD, termed "lysosomal macular dystrophy." This specific group of disorders may share molecular pathology mechanisms with other LSDs, adaptinopathies, and potentially other forms of retinal blindness, suggesting the possibility of adopting common therapeutic strategies in the future.

Data and code availability

All variants identified in this study have been submitted to the ClinVar database (https://www.ncbi.nlm.nih.gov/clinvar/).

Acknowledgments

We would like to acknowledge all participants and their families for their involvement in this study. We extend our deepest gratitude to Patricia Galliker for her exceptional support in culturing the iPSC-RPE cells, which was instrumental to this work. We are also grateful to Virginie G. Peter, Patricia Späni, Raquel Rodrigues, Nils Schaerer, Daniela Hauenstein, Petra Rossouw, Chrysoula Gabrani, Dhryata Kamdar, Pierre Balmer, Mattias Van Heetvelde, Marieke De Bruyne, Inmaculada Martin-Merida, Lidia Rodríguez Peña, Luisa Quintanilla Mata, Esther Simarro Rueda, and Vasileia Maniadi for their support in collecting the data and to Sitta Föhr for finalizing the submission of the manuscript.

We thank the Imaging Core Facility (IMCF, Biozentrum, University of Basel), and in particular Alexia Loynton-Ferrand, for the technical assistance provided on the Stellaris 8 Falcon point scanning confocal microscope.

This research was made possible through access to data in the National Genomic Research Library, which is managed by Genomics England Limited (a wholly owned company of the Department of Health and Social Care). The National Genomic Research Library holds data provided by patients and collected by the NHS as part of their care and data collected as part of their participation in research. The National Genomic Research Library is funded by the National Institute for Health Research and NHS England. The Wellcome Trust, Cancer Research UK, and the Medical Research Council have also funded research infrastructure.

Part of Figure S4 and the graphical abstract were created with BioRender (www.biorender.com).

Funding sources are listed as supplemental information.

Author contributions

K.K., M.Q., and C.R. designed the study. K.K. and A.R.M. generated wet-lab experimental data. A.P.M. and A.R.M. collected human RPE tissue. K.K., F.C., M.Q., M.B., S.L., T.H., P.B.-M., A.A.F., L.F.-C., I.P.-R., G.G.G., D.S., P.M., T.Z., K.S., T.B.H., C.A., J.M.M., D.S., T.B.-Y., E.D.B., G.A., P.I.S., S.K., and C.R. were involved in the genetic data generation and analysis. M.Q. was responsible for computer-assisted analyses. L.J.-K., L. Kuehlewein, P.D.A., R.S., F.V.d.B., J.J., S.V., F.T., N.F., L. Koutroumanou, G.P., A.C.B., S.M., L.G., E.B., D.S., A.B.S., L.C.S., B.P.L., S.L., O.A.M., S.A., H.P.N.S., M.P., M.K.T., V.V., H.V.T., A.R.W., and C.S. contributed with collection and evaluation of clinical data. K.K., C.S., and C.R. wrote the original draft. All authors reviewed and approved the manuscript.

Declaration of interests

The authors declare no competing financial or non-financial interests.

Declaration of generative AI and AI-assisted technologies in the writing process

AI technology was used to check for spelling mistakes and, in general, copy-edit parts of our manuscript draft. No new text was generated by using AI tools.

Supplemental information

Supplemental information can be found online at https://doi.org/10.1016/j.ajhg.2025.02.015.

Web resources

ClinVar, https://www.ncbi.nlm.nih.gov/clinvar/
Franklin, https://franklin.genoox.com
GeniE, https://genie.broadinstitute.org/
gnomAD, https://gnomad.broadinstitute.org/
OMIM, https://omim.org/
RetNet, http://retnet.org

Received: September 12, 2024
Accepted: February 13, 2025
Published: March 12, 2025

References

1. Schneider, N., Sundaresan, Y., Gopalakrishnan, P., Beryozkin, A., Hanany, M., Levanon, E.Y., Banin, E., Ben-Aroya, S., and Sharon, D. (2022). Inherited retinal diseases: Linking genes, disease-causing variants, and relevant therapeutic modalities. Prog. Retin. Eye Res. *89*, 101029.
2. Rahman, N., Georgiou, M., Khan, K.N., and Michaelides, M. (2020). Macular dystrophies: clinical and imaging features, molecular genetics and therapeutic options. Br. J. Ophthalmol. *104*, 451–460.
3. Perea-Romero, I., Gordo, G., Iancu, I.F., Del Pozo-Valero, M., Almoguera, B., Blanco-Kelly, F., Carreño, E., Jimenez-Rolando, B., Lopez-Rodriguez, R., Lorda-Sanchez, I., et al. (2021). Genetic landscape of 6089 inherited retinal dystrophies affected cases in Spain and their therapeutic and extended epidemiological implications. Sci. Rep. *11*, 1526.
4. Dockery, A., Whelan, L., Humphries, P., and Farrar, G.J. (2021). Next-Generation Sequencing Applications for Inherited Retinal Diseases. Int. J. Mol. Sci. *22*, 5684.
5. Stone, E.M., Andorf, J.L., Whitmore, S.S., DeLuca, A.P., Giacalone, J.C., Streb, L.M., Braun, T.A., Mullins, R.F., Scheetz, T.E., Sheffield, V.C., and Tucker, B.A. (2017). Clinically

Focused Molecular Investigation of 1000 Consecutive Families with Inherited Retinal Disease. Ophthalmology *124*, 1314–1331.
6. Pontikos, N., Arno, G., Jurkute, N., Schiff, E., Ba-Abbad, R., Malka, S., Gimenez, A., Georgiou, M., Wright, G., Armengol, M., et al. (2020). Genetic Basis of Inherited Retinal Disease in a Molecularly Characterized Cohort of More Than 3000 Families from the United Kingdom. Ophthalmology *127*, 1384–1394.
7. Karali, M., Testa, F., Di Iorio, V., Torella, A., Zeuli, R., Scarpato, M., Romano, F., Onore, M.E., Pizzo, M., Melillo, P., et al. (2022). Genetic epidemiology of inherited retinal diseases in a large patient cohort followed at a single center in Italy. Sci. Rep. *12*, 20815.
8. Peter, V.G., Kaminska, K., Santos, C., Quinodoz, M., Cancellieri, F., Cisarova, K., Pescini Gobert, R., Rodrigues, R., Custódio, S., Paris, L.P., et al. (2023). The first genetic landscape of inherited retinal dystrophies in Portuguese patients identifies recurrent homozygous mutations as a frequent cause of pathogenesis. PNAS Nexus *2*, pgad043.
9. Weisschuh, N., Mazzola, P., Zuleger, T., Schaeferhoff, K., Kühlewein, L., Kortüm, F., Witt, D., Liebmann, A., Falb, R., Pohl, L., et al. (2024). Diagnostic genome sequencing improves diagnostic yield: a prospective single-centre study in 1000 patients with inherited eye diseases. J. Med. Genet. *61*, 186–195.
10. Daich Varela, M., Bellingham, J., Motta, F., Jurkute, N., Ellingford, J.M., Quinodoz, M., Oprych, K., Niblock, M., Janeschitz-Kriegl, L., Kaminska, K., et al. (2023). Multidisciplinary team directed analysis of whole genome sequencing reveals pathogenic non-coding variants in molecularly undiagnosed inherited retinal dystrophies. Hum. Mol. Genet. *32*, 595–607.
11. Han, J.H., Rodenburg, K., Hayman, T., Calzetti, G., Kaminska, K., Quinodoz, M., Marra, M., Wallerich, S., Allon, G., Nagy, Z.Z., et al. (2024). Loss-of-function variants in UBAP1L cause autosomal recessive retinal degeneration. Genet. Med. *26*, 101106.
12. Bauwens, M., Celik, E., Zur, D., Lin, S., Quinodoz, M., Michaelides, M., Webster, A.R., Van Den Broeck, F., Leroy, B.P., Rizel, L., et al. (2024). Mutations in SAMD7 cause autosomal-recessive macular dystrophy with or without cone dysfunction. Am. J. Hum. Genet. *111*, 393–402.
13. Nuzhat, N., Van Schil, K., Liakopoulos, S., Bauwens, M., Rey, A.D., Käseberg, S., Jäger, M., Willer, J.R., Winter, J., Truong, H.M., et al. (2023). CEP162 deficiency causes human retinal degeneration and reveals a dual role in ciliogenesis and neurogenesis. J. Clin. Investig. *133*, e161156.
14. Millo, T., Rivera, A., Obolensky, A., Marks-Ohana, D., Xu, M., Li, Y., Wilhelm, E., Gopalakrishnan, P., Gross, M., Rosin, B., et al. (2022). Identification of autosomal recessive novel genes and retinal phenotypes in members of the solute carrier (SLC) superfamily. Genet. Med. *24*, 1523–1535.
15. Platt, F.M., d'Azzo, A., Davidson, B.L., Neufeld, E.F., and Tifft, C.J. (2018). Lysosomal storage diseases. Nat. Rev. Dis. Primers *4*, 27.
16. Wang, F., Wang, H., Tuan, H.F., Nguyen, D.H., Sun, V., Keser, V., Bowne, S.J., Sullivan, L.S., Luo, H., Zhao, L., et al. (2014). Next generation sequencing-based molecular diagnosis of retinitis pigmentosa: identification of a novel genotype-phenotype correlation and clinical refinements. Hum. Genet. *133*, 331–345.
17. Haer-Wigman, L., Newman, H., Leibu, R., Bax, N.M., Baris, H.N., Rizel, L., Banin, E., Massarweh, A., Roosing, S., Lefeber, D.J., et al. (2015). Non-syndromic retinitis pigmentosa due to mutations in the mucopolysaccharidosis type IIIC gene, heparan-alpha-glucosaminide N-acetyltransferase (HGSNAT). Hum. Mol. Genet. *24*, 3742–3751.
18. Roosing, S., van den Born, L.I., Sangermano, R., Banfi, S., Koenekoop, R.K., Zonneveld-Vrieling, M.N., Klaver, C.C.W., van Lith-Verhoeven, J.J.C., Cremers, F.P.M., den Hollander, A.I., and Hoyng, C.B. (2015). Mutations in MFSD8, encoding a lysosomal membrane protein, are associated with nonsyndromic autosomal recessive macular dystrophy. Ophthalmology *122*, 170–179.
19. Bauwens, M., Storch, S., Weisschuh, N., Ceuterick-de Groote, C., De Rycke, R., Guillemyn, B., De Jaegere, S., Coppieters, F., Van Coster, R., Leroy, B.P., and De Baere, E. (2020). Functional characterization of novel MFSD8 pathogenic variants anticipates neurological involvement in juvenile isolated maculopathy. Clin. Genet. *97*, 426–436.
20. Tang, C., Han, J., Dalvi, S., Manian, K., Winschel, L., Volland, S., Soto, C.A., Galloway, C.A., Spencer, W., Roll, M., et al. (2021). A human model of Batten disease shows role of CLN3 in phagocytosis at the photoreceptor-RPE interface. Commun. Biol. *4*, 161.
21. Hirst, J., Itzhak, D.N., Antrobus, R., Borner, G.H.H., and Robinson, M.S. (2018). Role of the AP-5 adaptor protein complex in late endosome-to-Golgi retrieval. PLoS Biol. *16*, e2004411.
22. Hirst, J., Hesketh, G.G., Gingras, A.C., and Robinson, M.S. (2021). Rag GTPases and phosphatidylinositol 3-phosphate mediate recruitment of the AP-5/SPG11/SPG15 complex. J. Cell Biol. *220*, e202002075.
23. Hirst, J., Barlow, L.D., Francisco, G.C., Sahlender, D.A., Seaman, M.N.J., Dacks, J.B., and Robinson, M.S. (2011). The fifth adaptor protein complex. PLoS Biol. *9*, e1001170.
24. Hamosh, A., Scott, A.F., Amberger, J., Valle, D., and McKusick, V.A. (2000). Online Mendelian Inheritance in Man (OMIM). Hum. Mutat. *15*, 57–61.
25. Hirst, J., Edgar, J.R., Esteves, T., Darios, F., Madeo, M., Chang, J., Roda, R.H., Dürr, A., Anheim, M., Gellera, C., et al. (2015). Loss of AP-5 results in accumulation of aberrant endolysosomes: defining a new type of lysosomal storage disease. Hum. Mol. Genet. *24*, 4984–4996.
26. Perea-Romero, I., Blanco-Kelly, F., Sanchez-Navarro, I., Lorda-Sanchez, I., Tahsin-Swafiri, S., Avila-Fernandez, A., Martin-Merida, I., Trujillo-Tiebas, M.J., Lopez-Rodriguez, R., Rodriguez de Alba, M., et al. (2021). NGS and phenotypic ontology-based approaches increase the diagnostic yield in syndromic retinal diseases. Hum. Genet. *140*, 1665–1678.
27. Hayman, T., Millo, T., Hendler, K., Chowers, I., Gross, M., Banin, E., and Sharon, D. (2024). Whole exome sequencing of 491 individuals with inherited retinal diseases reveals a large spectrum of variants and identification of novel candidate genes. J. Med. Genet. *61*, 224–231.
28. Del Pozo-Valero, M., Almoallem, B., Dueñas Rey, A., Mahieu, Q., Van Heetvelde, M., Jeddawi, L., Bauwens, M., and De Baere, E. (2024). Autozygome-guided exome-first study in a consanguineous cohort with early-onset retinal disease uncovers an isolated RIMS2 phenotype and a retina-enriched RIMS2 isoform. Clin. Genet. *106*, 127–139.
29. Caulfield, M., Davies, J., Dennys, M., Elbahy, L., Fowler, T., Hill, S., Hubbard, T., Jostins, L., Maltby, N., Mahon-Pearson, J., et al. (2020). National Genomic Research Library. DOI: https://doi.org/10.6084/m9.figshare.4530893.v7.
30. 100,000 Genomes Project Pilot Investigators, Smedley, D., Smith, K.R., Martin, A., Thomas, E.A., McDonagh, E.M.,

Cipriani, V., Ellingford, J.M., Arno, G., and Tucci, A. (2021). 100,000 Genomes Pilot on Rare-Disease Diagnosis in Health Care - Preliminary Report. N. Engl. J. Med. *385*, 1868–1880.

31. Martin, A.R., Williams, E., Foulger, R.E., Leigh, S., Daugherty, L.C., Niblock, O., Leong, I.U.S., Smith, K.R., Gerasimenko, O., Haraldsdottir, E., et al. (2019). PanelApp crowdsources expert knowledge to establish consensus diagnostic gene panels. Nat. Genet. *51*, 1560–1565.
32. Freeman, P.J., Hart, R.K., Gretton, L.J., Brookes, A.J., and Dalgleish, R. (2018). VariantValidator: Accurate validation, mapping, and formatting of sequence variation descriptions. Hum. Mutat. *39*, 61–68.
33. den Dunnen, J.T., Dalgleish, R., Maglott, D.R., Hart, R.K., Greenblatt, M.S., McGowan-Jordan, J., Roux, A.F., Smith, T., Antonarakis, S.E., and Taschner, P.E.M. (2016). HGVS Recommendations for the Description of Sequence Variants: 2016 Update. Hum. Mutat. *37*, 564–569.
34. Richards, S., Aziz, N., Bale, S., Bick, D., Das, S., Gastier-Foster, J., Grody, W.W., Hegde, M., Lyon, E., Spector, E., et al. (2015). Standards and guidelines for the interpretation of sequence variants: a joint consensus recommendation of the American College of Medical Genetics and Genomics and the Association for Molecular Pathology. Genet. Med. *17*, 405–424.
35. Riggs, E.R., Andersen, E.F., Cherry, A.M., Kantarci, S., Kearney, H., Patel, A., Raca, G., Ritter, D.I., South, S.T., Thorland, E.C., et al. (2020). Technical standards for the interpretation and reporting of constitutional copy-number variants: a joint consensus recommendation of the American College of Medical Genetics and Genomics (ACMG) and the Clinical Genome Resource (ClinGen). Genet. Med. *22*, 245–257.
36. Karczewski, K.J., Francioli, L.C., Tiao, G., Cummings, B.B., Alföldi, J., Wang, Q., Collins, R.L., Laricchia, K.M., Ganna, A., Birnbaum, D.P., et al. (2020). The mutational constraint spectrum quantified from variation in 141,456 humans. Nature *581*, 434–443.
37. Robinson, J.T., Thorvaldsdóttir, H., Winckler, W., Guttman, M., Lander, E.S., Getz, G., and Mesirov, J.P. (2011). Integrative genomics viewer. Nat. Biotechnol. *29*, 24–26.
38. Quinodoz, M., Kaminska, K., Cancellieri, F., Han, J.H., Peter, V.G., Celik, E., Janeschitz-Kriegl, L., Schärer, N., Hauenstein, D., György, B., et al. (2024). Detection of elusive DNA copy-number variations in hereditary disease and cancer through the use of noncoding and off-target sequencing reads. Am. J. Hum. Genet. *111*, 701–713.
39. Roller, E., Ivakhno, S., Lee, S., Royce, T., and Tanner, S. (2016). Canvas: versatile and scalable detection of copy number variants. Bioinformatics *32*, 2375–2377.
40. Chen, X., Schulz-Trieglaff, O., Shaw, R., Barnes, B., Schlesinger, F., Källberg, M., Cox, A.J., Kruglyak, S., and Saunders, C.T. (2016). Manta: rapid detection of structural variants and indels for germline and cancer sequencing applications. Bioinformatics *32*, 1220–1222.
41. Di Tommaso, P., Moretti, S., Xenarios, I., Orobitg, M., Montanyola, A., Chang, J.M., Taly, J.F., and Notredame, C. (2011). T-Coffee: a web server for the multiple sequence alignment of protein and RNA sequences using structural information and homology extension. Nucleic Acids Res. *39*, W13–W17.
42. Armougom, F., Moretti, S., Poirot, O., Audic, S., Dumas, P., Schaeli, B., Keduas, V., and Notredame, C. (2006). Expresso: automatic incorporation of structural information in multiple sequence alignments using 3D-Coffee. Nucleic Acids Res. *34*, W604–W608.
43. Jumper, J., Evans, R., Pritzel, A., Green, T., Figurnov, M., Ronneberger, O., Tunyasuvunakool, K., Bates, R., Žídek, A., Potapenko, A., et al. (2021). Highly accurate protein structure prediction with AlphaFold. Nature *596*, 583–589.
44. Untergasser, A., Nijveen, H., Rao, X., Bisseling, T., Geurts, R., and Leunissen, J.A.M. (2007). Primer3Plus, an enhanced web interface to Primer3. Nucleic Acids Res. *35*, W71–W74.
45. Hunt, S.E., McLaren, W., Gil, L., Thormann, A., Schuilenburg, H., Sheppard, D., Parton, A., Armean, I.M., Trevanion, S.J., Flicek, P., and Cunningham, F. (2018). Ensembl variation resources. Database *2018*, bay119.
46. Kuwahara, A., Ozone, C., Nakano, T., Saito, K., Eiraku, M., and Sasai, Y. (2015). Generation of a ciliary margin-like stem cell niche from self-organizing human retinal tissue. Nat. Commun. *6*, 6286.
47. Tagawa, M., Ikeda, H.O., Hata, M., Inoue, Y., Iwai, S., and Tsujikawa, A. (2022). A Protocol for Stepwise Differentiation of Induced Pluripotent Stem Cells into Retinal Pigment Epithelium. Methods Mol. Biol. *2549*, 307–320.
48. Landrum, M.J., Lee, J.M., Benson, M., Brown, G., Chao, C., Chitipiralla, S., Gu, B., Hart, J., Hoffman, D., Hoover, J., et al. (2016). ClinVar: public archive of interpretations of clinically relevant variants. Nucleic Acids Res. *44*, D862–D868.
49. Hanany, M., Rivolta, C., and Sharon, D. (2020). Worldwide carrier frequency and genetic prevalence of autosomal recessive inherited retinal diseases. Proc. Natl. Acad. Sci. USA *117*, 2710–2716.
50. Li, C.H.Z., Pas, J.A.A.H., Corradi, Z., Hitti-Malin, R.J., Hoogstede, A., Runhart, E.H., Dhooge, P.P.A., Collin, R.W.J., Cremers, F.P.M., and Hoyng, C.B. (2024). Study of Late-Onset Stargardt Type 1 Disease: Characteristics, Genetics, and Progression. Ophthalmology *131*, 87–97.
51. Boon, C.J.F., Klevering, B.J., Cremers, F.P.M., Zonneveld-Vrieling, M.N., Theelen, T., Den Hollander, A.I., and Hoyng, C.B. (2009). Central areolar choroidal dystrophy. Ophthalmology *116*, 771–782.e1.
52. Singh Grewal, S., Smith, J.J., and Carr, A.J.F. (2021). Bestrophinopathies: perspectives on clinical disease, Bestrophin-1 function and developing therapies. Ther. Adv. Ophthalmol. *13*, 2515841421997191.
53. Beryozkin, A., Sher, I., Ehrenberg, M., Zur, D., Newman, H., Gradstein, L., Simaan, F., Rotenstreich, Y., Goldenberg-Cohen, N., Bahar, I., et al. (2024). Best Disease: Global Mutations Review, Genotype-Phenotype Correlation, and Prevalence Analysis in the Israeli Population. Investig. Ophthalmol. Vis. Sci. *65*, 39.
54. Bandah-Rozenfeld, D., Littink, K.W., Ben-Yosef, T., Strom, T.M., Chowers, I., Collin, R.W.J., den Hollander, A.I., van den Born, L.I., Zonneveld, M.N., Merin, S., et al. (2010). Novel null mutations in the EYS gene are a frequent cause of autosomal recessive retinitis pigmentosa in the Israeli population. Investig. Ophthalmol. Vis. Sci. *51*, 4387–4394.
55. Weil, D., El-Amraoui, A., Masmoudi, S., Mustapha, M., Kikkawa, Y., Lainé, S., Delmaghani, S., Adato, A., Nadifi, S., Zina, Z.B., et al. (2003). Usher syndrome type I G (USH1G) is caused by mutations in the gene encoding SANS, a protein that associates with the USH1C protein, harmonin. Hum. Mol. Genet. *12*, 463–471.
56. Quinodoz, M., Peter, V.G., Bedoni, N., Royer Bertrand, B., Cisarova, K., Salmaninejad, A., Sepahi, N., Rodrigues, R., Piran,

M., Mojarrad, M., et al. (2021). AutoMap is a high performance homozygosity mapping tool using next-generation sequencing data. Nat. Commun. *12*, 518.
57. Jaganathan, K., Kyriazopoulou Panagiotopoulou, S., McRae, J.F., Darbandi, S.F., Knowles, D., Li, Y.I., Kosmicki, J.A., Arbelaez, J., Cui, W., Schwartz, G.B., et al. (2019). Predicting Splicing from Primary Sequence with Deep Learning. Cell *176*, 535–548.e24.
58. Slabicki, M., Theis, M., Krastev, D.B., Samsonov, S., Mundwiller, E., Junqueira, M., Paszkowski-Rogacz, M., Teyra, J., Heninger, A.K., Poser, I., et al. (2010). A genome-scale DNA repair RNAi screen identifies SPG48 as a novel gene associated with hereditary spastic paraplegia. PLoS Biol. *8*, e1000408.
59. Quinodoz, M., Peter, V.G., Cisarova, K., Royer-Bertrand, B., Stenson, P.D., Cooper, D.N., Unger, S., Superti-Furga, A., and Rivolta, C. (2022). Analysis of missense variants in the human genome reveals widespread gene-specific clustering and improves prediction of pathogenicity. Am. J. Hum. Genet. *109*, 457–470.
60. Ebrahimi-Fakhari, D., Teinert, J., Behne, R., Wimmer, M., D'Amore, A., Eberhardt, K., Brechmann, B., Ziegler, M., Jensen, D.M., Nagabhyrava, P., et al. (2020). Defining the clinical, molecular and imaging spectrum of adaptor protein complex 4-associated hereditary spastic paraplegia. Brain *143*, 2929–2944.
61. Jian, X., Boerwinkle, E., and Liu, X. (2014). In silico prediction of splice-altering single nucleotide variants in the human genome. Nucleic Acids Res. *42*, 13534–13544.
62. Danis, D., Jacobsen, J.O.B., Carmody, L.C., Gargano, M.A., McMurry, J.A., Hegde, A., Haendel, M.A., Valentini, G., Smedley, D., and Robinson, P.N. (2021). Interpretable prioritization of splice variants in diagnostic next-generation sequencing. Am. J. Hum. Genet. *108*, 2205.
63. Yeo, G., and Burge, C.B. (2004). Maximum entropy modeling of short sequence motifs with applications to RNA splicing signals. J. Comput. Biol. *11*, 377–394.
64. Shamsani, J., Kazakoff, S.H., Armean, I.M., McLaren, W., Parsons, M.T., Thompson, B.A., O'Mara, T.A., Hunt, S.E., Waddell, N., and Spurdle, A.B. (2019). A plugin for the Ensembl Variant Effect Predictor that uses MaxEntScan to predict variant spliceogenicity. Bioinformatics *35*, 2315–2317.
65. Breza, M., Hirst, J., Chelban, V., Banneau, G., Tissier, L., Kol, B., Bourinaris, T., Said, S.A., Péréon, Y., Heinzmann, A., et al. (2021). Expanding the Spectrum of AP5Z1-Related Hereditary Spastic Paraplegia (HSP-SPG48): A Multicenter Study on a Rare Disease. Mov. Disord. *36*, 1034–1038.
66. Khundadze, M., Ribaudo, F., Hussain, A., Rosentreter, J., Nietzsche, S., Thelen, M., Winter, D., Hoffmann, B., Afzal, M.A., Hermann, T., et al. (2019). A mouse model for SPG48 reveals a block of autophagic flux upon disruption of adaptor protein complex five. Neurobiol. Dis. *127*, 419–431.
67. Hirst, J., Irving, C., and Borner, G.H.H. (2013). Adaptor protein complexes AP-4 and AP-5: new players in endosomal trafficking and progressive spastic paraplegia. Traffic *14*, 153–164.
68. Hirst, J., Borner, G.H.H., Edgar, J., Hein, M.Y., Mann, M., Buchholz, F., Antrobus, R., and Robinson, M.S. (2013). Interaction between AP-5 and the hereditary spastic paraplegia proteins SPG11 and SPG15. Mol. Biol. Cell *24*, 2558–2569.
69. Saksens, N.T.M., Fleckenstein, M., Schmitz-Valckenberg, S., Holz, F.G., den Hollander, A.I., Keunen, J.E.E., Boon, C.J.F., and Hoyng, C.B. (2014). Macular dystrophies mimicking age-related macular degeneration. Prog. Retin. Eye Res. *39*, 23–57.
70. Hirst, J., Madeo, M., Smets, K., Edgar, J.R., Schols, L., Li, J., Yarrow, A., Deconinck, T., Baets, J., Van Aken, E., et al. (2016). Complicated spastic paraplegia in patients with AP5Z1 mutations (SPG48). Neurol. Genet. *2*, e98.
71. de Freitas, J.L., Rezende Filho, F.M., Sallum, J.M.F., França, M.C., Jr., Pedroso, J.L., and Barsottini, O.G.P. (2020). Ophthalmological changes in hereditary spastic paraplegia and other genetic diseases with spastic paraplegia. J. Neurol. Sci. *409*, 116620.
72. Jin, P., Wang, Y., Nian, N., Wang, G.Q., and Fu, X.M. (2023). Hereditary spastic paraplegia (SPG 48) with deafness and azoospermia: A case report. Front. Neurol. *14*, 1156100.
73. Schiff, E.R., Daich Varela, M., Robson, A.G., Pierpoint, K., Ba-Abbad, R., Nutan, S., Zein, W.M., Ullah, E., Huryn, L.A., Tuupanen, S., et al. (2020). A genetic and clinical study of individuals with nonsyndromic retinopathy consequent upon sequence variants in HGSNAT, the gene associated with Sanfilippo C mucopolysaccharidosis. Am. J. Med. Genet. C Semin. Med. Genet. *184*, 631–643.
74. Kolesnikova, M., Lima de Carvalho, J.R., Jr., Oh, J.K., Soucy, M., Demirkol, A., Kim, A.H., Tsang, S.H., and Breazzano, M.P. (2023). Phenotypic Variability of Retinal Disease Among a Cohort of Patients With Variants in the CLN Genes. Investig. Ophthalmol. Vis. Sci. *64*, 23.
75. Park, S.Y., and Guo, X. (2014). Adaptor protein complexes and intracellular transport. Biosci. Rep. *34*, e00123.
76. Shin, J., Nile, A., and Oh, J.W. (2021). Role of adaptin protein complexes in intracellular trafficking and their impact on diseases. Bioengineered *12*, 8259–8278.
77. Sanger, A., Hirst, J., Davies, A.K., and Robinson, M.S. (2019). Adaptor protein complexes and disease at a glance. J. Cell Sci. *132*, jcs222992.
78. Martinelli, D., and Dionisi-Vici, C. (2014). AP1S1 defect causing MEDNIK syndrome: a new adaptinopathy associated with defective copper metabolism. Ann. N. Y. Acad. Sci. *1314*, 55–63.
79. Burgueno-Montanes, C. (2022). Kjellin's syndrome: Spastic paraplegia and multifocal pattern dystrophy simulating fundus flavimaculatus. Arch. Soc. Esp. Oftalmol. *97*, 714–718.
80. Brock, V., Wissocq, A., Geoffre, N., Marks, C., Canel, V., Huin, V., and Smirnov, V.M. (2024). Late-onset Kjellin syndrome: Diagnosis of SPG11 on fundus examination. Eur. J. Ophthalmol. *34*, NP44–NP46.
81. Puech, B., Lacour, A., Stevanin, G., Sautiere, B.G., Devos, D., Depienne, C., Denis, E., Mundwiller, E., Ferriby, D., Vermersch, P., and Defoort-Dhellemmes, S. (2011). Kjellin syndrome: long-term neuro-ophthalmologic follow-up and novel mutations in the SPG11 gene. Ophthalmology *118*, 564–573.
82. Kjellin, K. (1959). Familial spastic paraplegia with amyotrophy, oligophrenia, and central retinal degeneration. Arch. Neurol. *1*, 133–140.
83. Renvoise, B., Chang, J., Singh, R., Yonekawa, S., FitzGibbon, E.J., Mankodi, A., Vanderver, A., Schindler, A., Toro, C., Gahl, W.A., et al. (2014). Lysosomal abnormalities in hereditary spastic paraplegia types SPG15 and SPG11. Ann. Clin. Transl. Neurol. *1*, 379–389.
84. Chang, J., Lee, S., and Blackstone, C. (2014). Spastic paraplegia proteins spastizin and spatacsin mediate autophagic lysosome reformation. J. Clin. Investig. *124*, 5249–5262.

85. Manley, A., Meshkat, B.I., Jablonski, M.M., and Hollingsworth, T.J. (2023). Cellular and Molecular Mechanisms of Pathogenesis Underlying Inherited Retinal Dystrophies. Biomolecules *13*, 271.
86. Thompson, D.A., Li, Y., McHenry, C.L., Carlson, T.J., Ding, X., Sieving, P.A., Apfelstedt-Sylla, E., and Gal, A. (2001). Mutations in the gene encoding lecithin retinol acyltransferase are associated with early-onset severe retinal dystrophy. Nat. Genet. *28*, 123–124.
87. Feng, W., Yasumura, D., Matthes, M.T., LaVail, M.M., and Vollrath, D. (2002). Mertk triggers uptake of photoreceptor outer segments during phagocytosis by cultured retinal pigment epithelial cells. J. Biol. Chem. *277*, 17016–17022.
88. Gal, A., Li, Y., Thompson, D.A., Weir, J., Orth, U., Jacobson, S.G., Apfelstedt-Sylla, E., and Vollrath, D. (2000). Mutations in MERTK, the human orthologue of the RCS rat retinal dystrophy gene, cause retinitis pigmentosa. Nat. Genet. *26*, 270–271.
89. Marmorstein, L.Y., Munier, F.L., Arsenijevic, Y., Schorderet, D.F., McLaughlin, P.J., Chung, D., Traboulsi, E., and Marmorstein, A.D. (2002). Aberrant accumulation of EFEMP1 underlies drusen formation in Malattia Leventinese and age-related macular degeneration. Proc. Natl. Acad. Sci. USA *99*, 13067–13072.
90. Keeling, E., Lotery, A.J., Tumbarello, D.A., and Ratnayaka, J.A. (2018). Impaired Cargo Clearance in the Retinal Pigment Epithelium (RPE) Underlies Irreversible Blinding Diseases. Cells *7*, 16.
91. Boya, P., Kaarniranta, K., Handa, J.T., and Sinha, D. (2023). Lysosomes in retinal health and disease. Trends Neurosci. *46*, 1067–1082.
92. Sinha, D., Valapala, M., Shang, P., Hose, S., Grebe, R., Lutty, G.A., Zigler, J.S., Jr., Kaarniranta, K., and Handa, J.T. (2016). Lysosomes: Regulators of autophagy in the retinal pigmented epithelium. Exp. Eye Res. *144*, 46–53.
93. Intartaglia, D., Giamundo, G., and Conte, I. (2022). Autophagy in the retinal pigment epithelium: a new vision and future challenges. FEBS J. *289*, 7199–7212.
94. Liu, J., Lu, W., Reigada, D., Nguyen, J., Laties, A.M., and Mitchell, C.H. (2008). Restoration of lysosomal pH in RPE cells from cultured human and ABCA4(-/-) mice: pharmacologic approaches and functional recovery. Investig. Ophthalmol. Vis. Sci. *49*, 772–780.
95. Zaydon, Y.A., and Tsang, S.H. (2024). The ABCs of Stargardt disease: the latest advances in precision medicine. Cell Biosci. *14*, 98.
96. Vantaggiato, C., Orso, G., Guarato, G., Brivio, F., Napoli, B., Panzeri, E., Masotti, S., Santorelli, F.M., Lamprou, M., Gumeni, S., et al. (2023). Rescue of lysosomal function as therapeutic strategy for SPG15 hereditary spastic paraplegia. Brain *146*, 1103–1120.
97. Buser, D.P., and Spang, A. (2023). Protein sorting from endosomes to the TGN. Front. Cell Dev. Biol. *11*, 1140605.

The American Journal of Human Genetics, Volume *112*

Supplemental information

Bi-allelic variants in three genes encoding distinct subunits of the vesicular AP-5 complex cause hereditary macular dystrophy

Karolina Kaminska, Francesca Cancellieri, Mathieu Quinodoz, Abigail R. Moye, Miriam Bauwens, Siying Lin, Lucas Janeschitz-Kriegl, Tamar Hayman, Pilar Barberán-Martínez, Regina Schlaeger, Filip Van den Broeck, Almudena Ávila Fernández, Lidia Fernández-Caballero, Irene Perea-Romero, Gema García-García, David Salom, Pascale Mazzola, Theresia Zuleger, Karin Poths, Tobias B. Haack, Julie Jacob, Sascha Vermeer, Frédérique Terbeek, Nicolas Feltgen, Alexandre P. Moulin, Louisa Koutroumanou, George Papadakis, Andrew C. Browning, Savita Madhusudhan, Lotta Gränse, Eyal Banin, Ana Berta Sousa, Luisa Coutinho Santos, Laura Kuehlewein, Pietro De Angeli, Bart P. Leroy, Omar A. Mahroo, Fay Sedgwick, James Eden, Maximilian Pfau, Sten Andréasson, Hendrik P.N. Scholl, Carmen Ayuso, José M. Millán, Dror Sharon, Miltiadis K. Tsilimbaris, Veronika Vaclavik, Hoai V. Tran, Tamar Ben-Yosef, Elfride De Baere, Andrew R. Webster, Gavin Arno, Panagiotis I. Sergouniotis, Susanne Kohl, Cristina Santos, and Carlo Rivolta

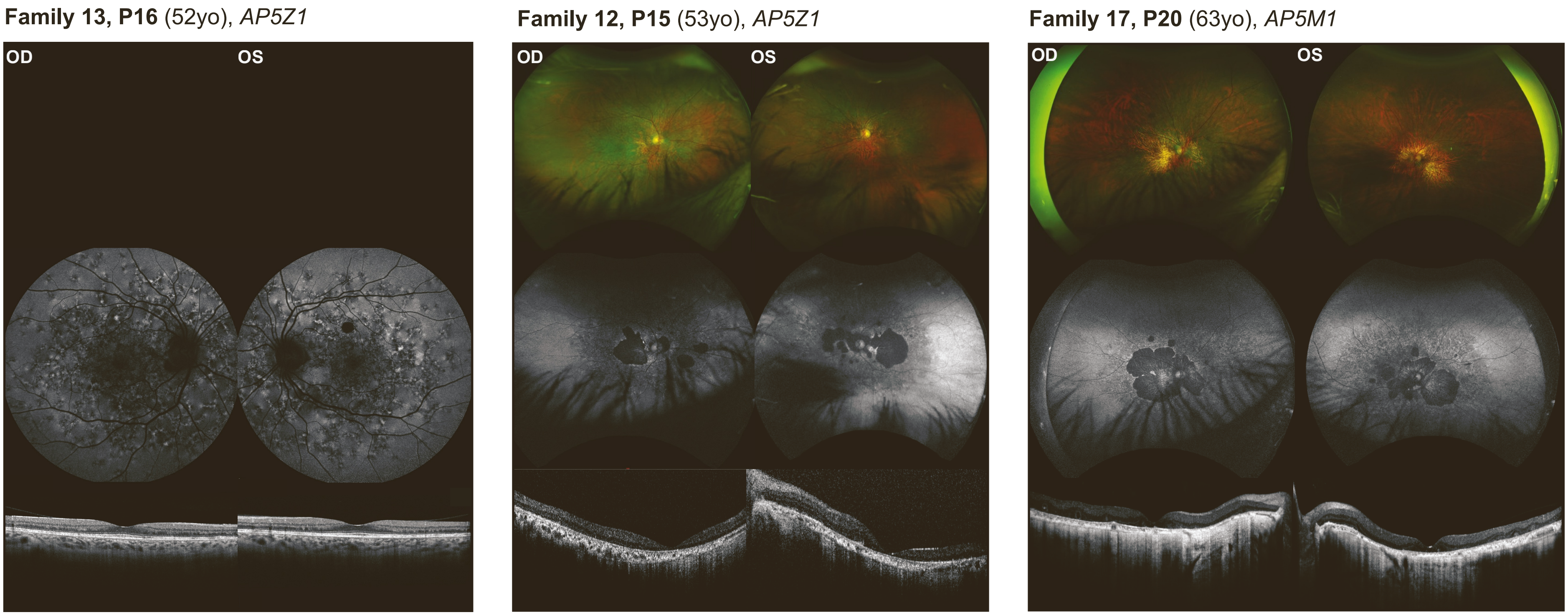

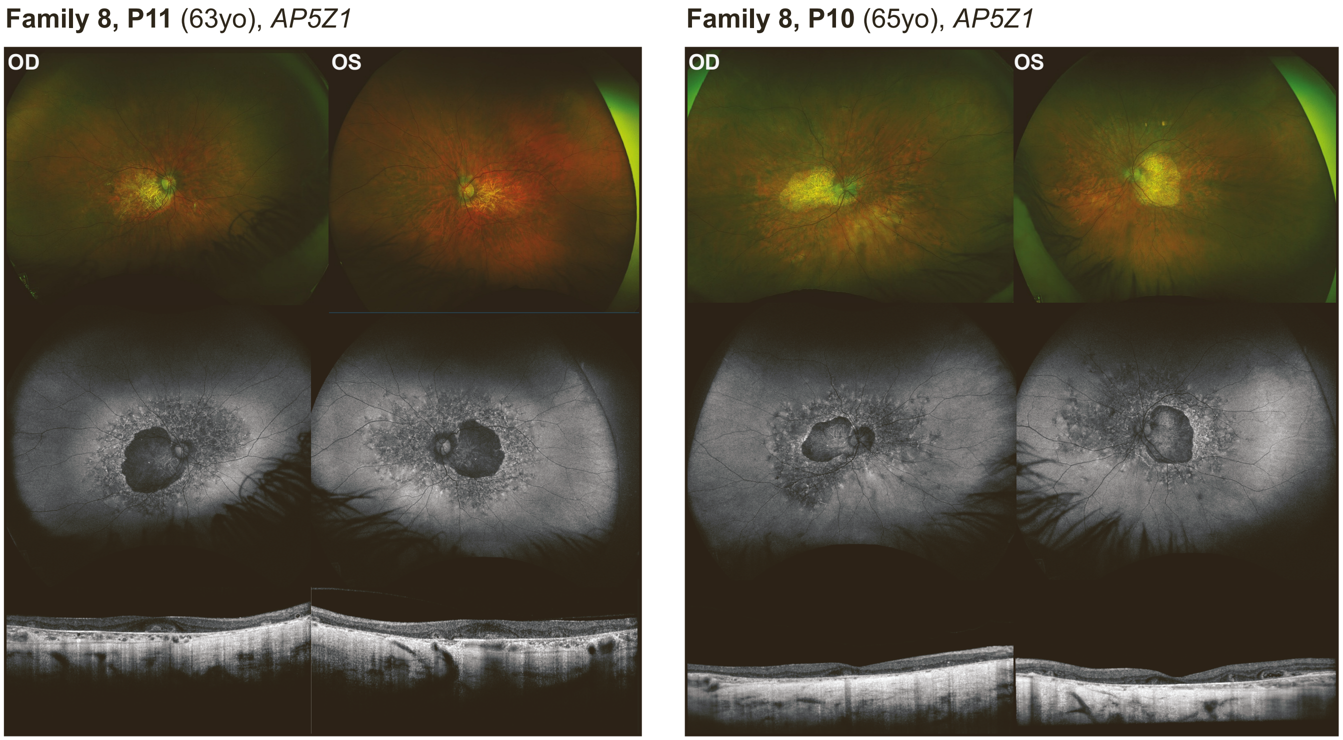

Figure S1: Multimodal retinal imaging of 5 additional individuals from this study. In each panel, the top row shows pseudocolor fundus images, the middle row fundus autofluorescence images (FAF), and the bottom row optical coherence tomography (OCT) scans. For individual P16 from Family 13, color fundus images were not available. This patient presented with a particular phenotype of early maculopathy without atrophy, at the age of 52 years. The other four cases (P15, age 53; P20, age 63; P11, age 63; and P10, age 65) had more advanced central chorioretinal atrophy, regardless of their genotypes. Images are ordered according to the patients' age. OD, right eye; OS, left eye. yo, years old.

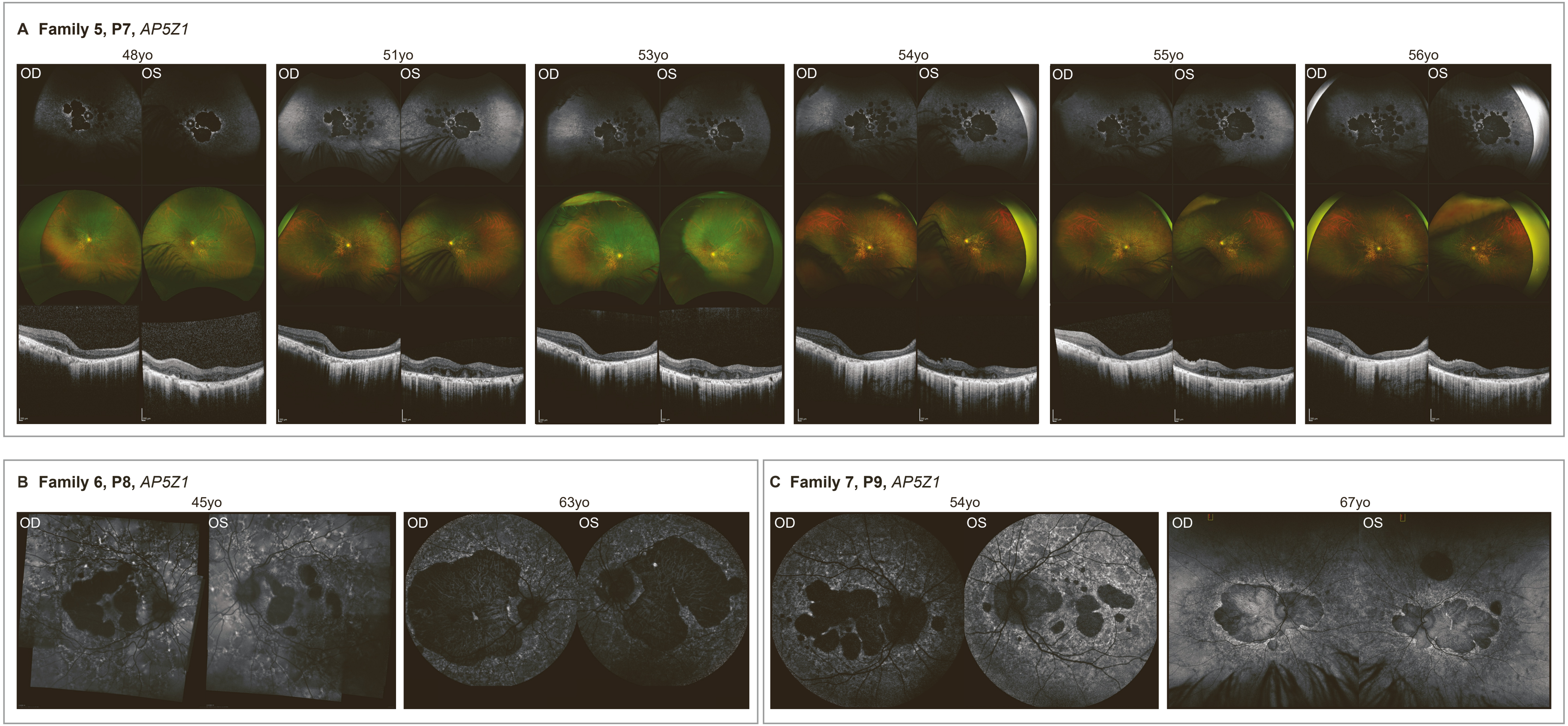

Figure S2: Longitudinal retinal imaging in 3 individuals, showing the progression of their disease. A. In each panel, the top row shows fundus autofluorescence images (FAF), the middle row pseudocolor fundus images, and the bottom row optical coherence tomography (OCT) scans for individual P7 from Family 5, at ages 48, 51, 53, 54, 55, and 56 years. B. FAF images of individual P8 from Family 6, taken at the ages of 45 and 63 years. C. FAF images of individual P9 from Family 7, at the age of 54 years, vs. widefield FAF from the same person at the age of 67 years. OD, right eye; OS, left eye. yo, years old.

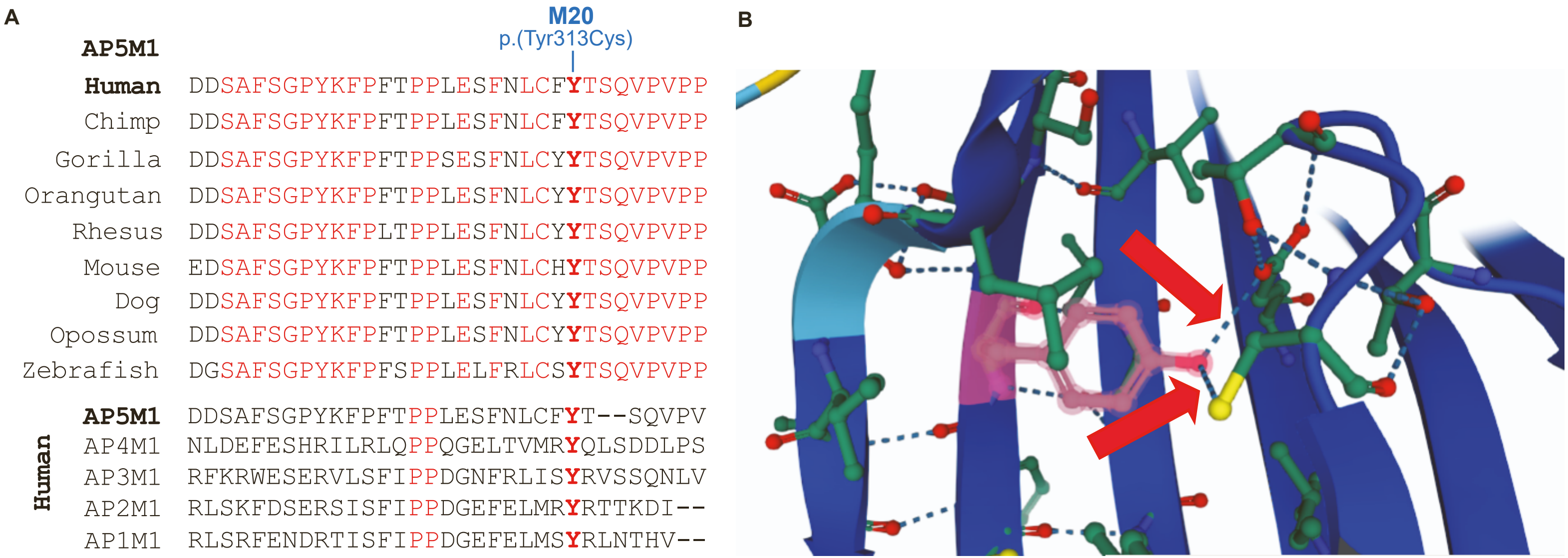

Figure S3: Properties of Tyr313 in AP5M1, affected by the M20 missense change. A. Alignment of part of AP5M1 with its orthologues from various vertebrates, as well as with its human paralogues. The Tyr313 amino acid residue (red, bold) is highly conserved, both across evolution and within the M components of all AP complexes. Other conserved amino acid residues are shown in red. B. Predicted 3D structure of the human AP5M1. The M20 missense [p.(Tyr313Cys)] replaces Tyr313 with a cysteine residue, abolishing the two hydrogen bonds that Tyr313 forms with Glu215 and Cys444 and likely destabilizing the native structure of AP5M1.

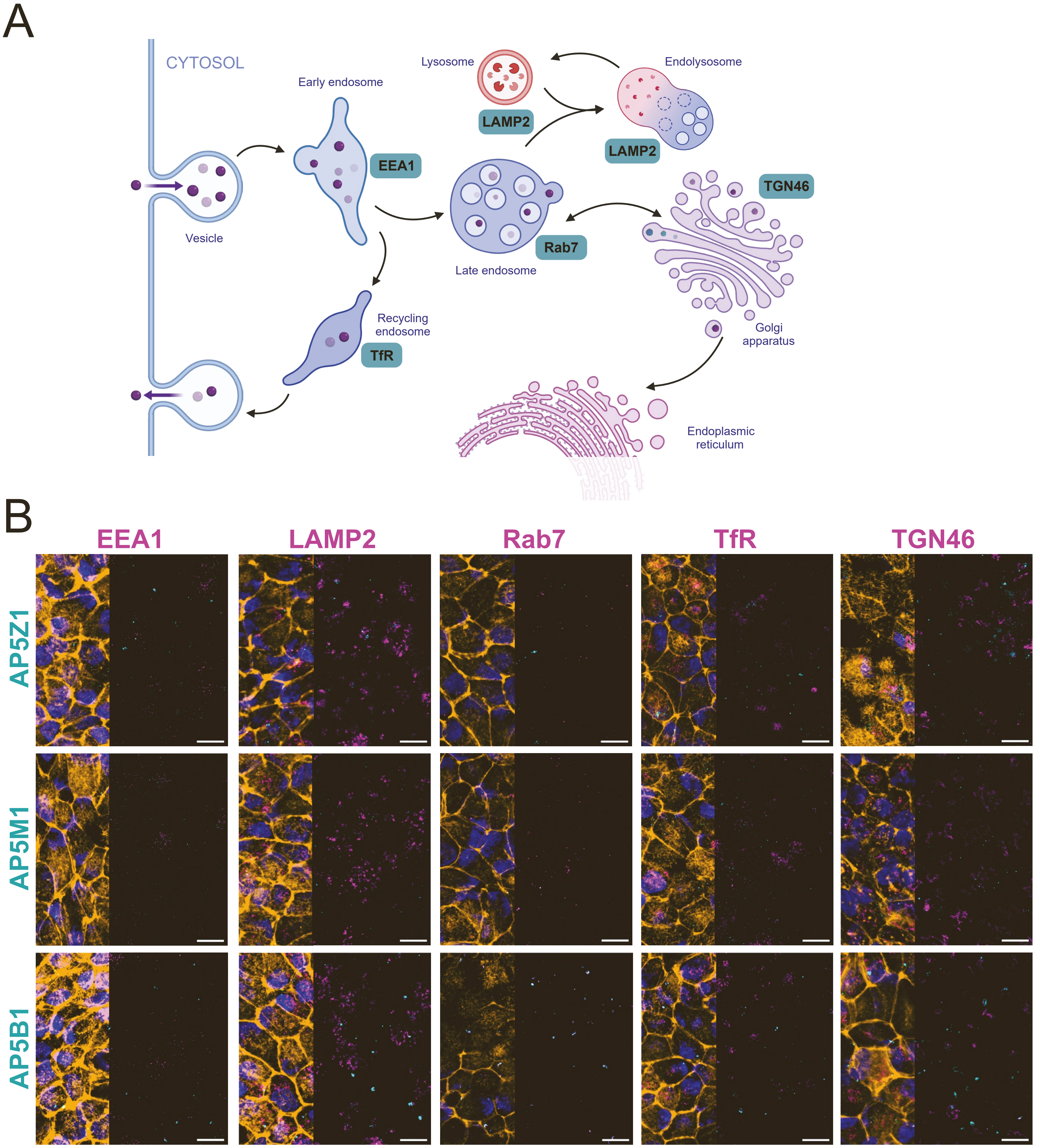

Figure S4: AP5Z1, AP5M1, and AP5B1 staining in human iPSC-RPE cell lines. A. Scheme showing intracellular vesicular transport, including relevant organelles and their associated markers used in this experiment. B. En face imaging of the iPSC-RPE monolayer sections co-stained with respective AP-5 subunits (AP5Z1, AP5M1, AP5B1, cyan) and markers of cellular compartments involved in the endolysosomal processing (magenta). AP-5 components co-localize with Rab7 and partially with TGN46, but not with the other markers. Signals from phalloidin (orange), labeling individual cells' plasma membranes, and DAPI (blue), staining nuclei, are filtered out from the right portion of each panel, to make the other markers more visible. Scale bars: 10 µm.

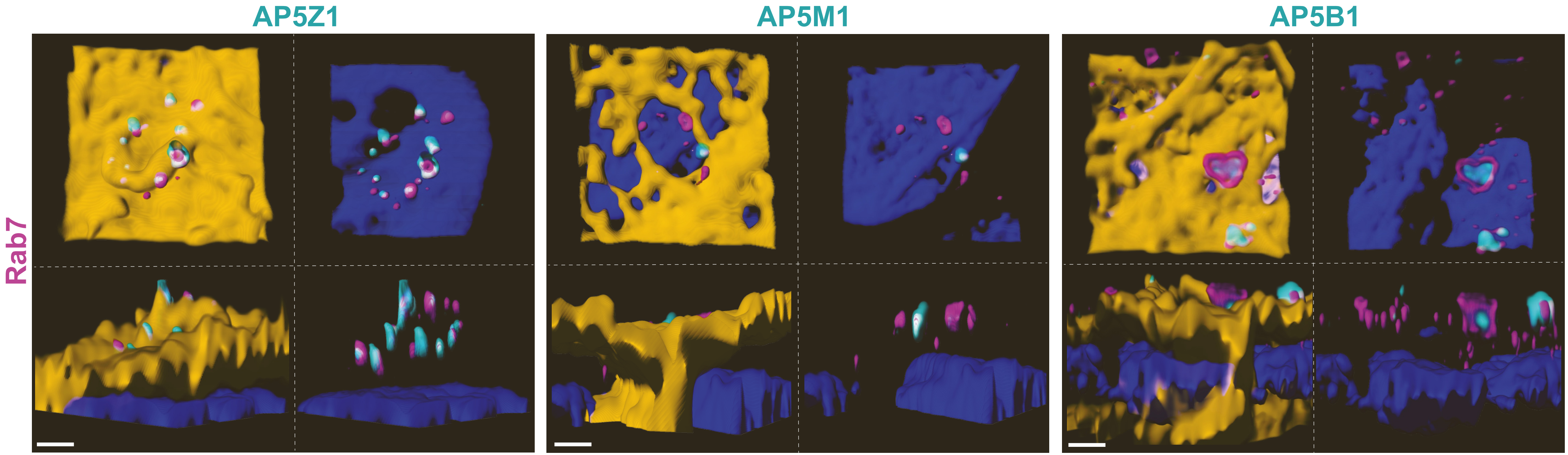

Figure S5: Spatial arrangement of AP5Z1, AP5M1, and AP5B1 in iPSC-RPE. Multi-dimensional visualization of the three studied AP-5 components (cyan) and Rab7-positive vesicles (magenta) in individual iPSC-RPE cells, with regions of their colocalization (white or cyan engulfed by magenta). The top row of each panel shows en-face images of the stained structures, with respect to actin filaments (labeled with **p**halloidin, yellow) and/or nuclei (marked with DAPI, blue). The bottom row of each panel displays cross-sectional views of the same images, illustrating the tubular morphology of these formations and their localization on the apical side of the RPE cells, potentially extending through the microvilli. Scale bars: 1µm.

Financial support

Funding was received from the following agencies: The Swiss National Science Foundation (grant No. 204285, to C.R.); Ministerio de Universidades (Grant No. FPU20/04736, to P.B.M.), Instituto de Salud Carlos III (ISCIII) and co-funded by the European Union (Grant No. CP22/00028 and Grant No. PI22/01371 to GGG, Grant No. PI22/00213, to J.M.M.); Ghent University Special Research Fund (Grant No. BOF20/GOA/023, to E.D.B. and B.P.L.), the European Joint Programme on Rare Diseases (Grant No. EJPRD19-234 Solve-RET, to E.D.B.), Fonds Wetenschappelijk Onderzoek (Grant No. FWO-1802220N, to E.D.B; Grant No. FWO-1803816N, to B.P.L.); the Israel Ministry of Health (Grant No. 3-18611, to T.B.-Y.); the Israel Science Foundation (Grant No. 1778/20- within the Israel Precision Medicine Partnership program, to D.S.); Grant No. PI22/00321, to C.A.; C.A. also receives support from the Centro de Investigación Biomédica en Red Enfermedades Raras (CIBERER, 06/07/0036), and IIS-FJD BioBank (PT13/0010/0012); OAM receives support from the Wellcome Trust and the NIHR Biomedical Research Centre at Moorfields Eye Hospital and the UCL Institute of Ophthalmology; G.A. is supported by a Fight for Sight UK early career investigator award (5045-5046), Moorfields Eye Charity (Stephen and Elizabeth Archer in memory of Marion Woods), NIH-P20GM139769 and the NIHR Biomedical Research Centre at Moorfields Eye Hopsital and the UCL Institute of Ophthalmology; T.B.H. received funding from the German Research Foundation (DFG; Grant No: 418081722 and 433158657) and the European Commission (Recon4IMD - GAP-101080997); P.I.S. receives support from the Wellcome Trust (224643/Z/21/Z) and the NIHR Manchester Biomedical Research Centre (NIHR203308). The views expressed are those of the authors and not necessarily those of the NIHR or the Department of Health and Social Care. E.D.B., B.P.L., S.K. are members of ERN-EYE (Framework Partnership Agreement No 739534-ERN-EYE). C.R., E.D.B., S.K. are members of the ProgRET network (HORIZON-MSCA-2022-DN, No.101120562).
